# Tumor suppressor genes and allele-specific expression: mechanisms and significance

**DOI:** 10.18632/oncotarget.27468

**Published:** 2020-01-28

**Authors:** Evan A. Clayton, Shareef Khalid, Dongjo Ban, Lu Wang, I. King Jordan, John F. McDonald

**Affiliations:** ^1^ Integrated Cancer Research Center, School of Biological Sciences, Georgia Institute of Technology, Atlanta, GA, USA; ^2^ School of Biological Sciences, Georgia Institute of Technology, Atlanta, GA, USA; ^3^ PanAmerican Bioinformatics Institute, Cali, Colombia; ^4^ Applied Bioinformatics Laboratory, Atlanta, GA, USA

**Keywords:** allele-specific expression, alternative splicing, antisense RNA, cancer, tumor-suppressor genes

## Abstract

Recent findings indicate that allele-specific expression (ASE) at specific cancer driver gene loci may be of importance in onset/progression of the disease. Of particular interest are loss-of-function (LOF) of tumor suppressor gene (TSGs) alleles. While LOF tumor suppressor mutations are typically considered to be recessive, if these mutant alleles can be significantly differentially expressed relative to wild-type alleles in heterozygotes, the clinical consequences could be significant.

LOF TSG alleles are shown to be segregating at high frequencies in world-wide populations of normal/healthy individuals. Matched sets of normal and tumor tissues isolated from 233 cancer patients representing four diverse tumor types demonstrate functionally important changes in patterns of ASE in individuals heterozygous for LOF TSG alleles associated with cancer onset/progression. While a variety of molecular mechanisms were identified as potentially contributing to changes in ASE patterns in cancer, changes in DNA copy number and allele-specific alternative splicing possibly mediated by antisense RNA emerged as predominant factors.

In conclusion, LOF TSGs are segregating in human populations at significant frequencies indicating that many otherwise healthy individuals are at elevated risk of developing cancer. Changes in ASE between normal and cancer tissues indicates that LOF TSG alleles may contribute to cancer onset/progression even when heterozygous with wild-type functional alleles.

## INTRODUCTION

The long-standing belief that cancer is a genetic disease driven by mutations in a select set of oncogenes and/or tumor suppressor genes (aka, “cancer driver” genes) [1–3], has been augmented in recent years to incorporate the auxiliary contribution of changes in a variety of regulatory controls [4–6]. Findings indicate that these additional regulatory controls may, in at least some instances, manifest as allele-specific expression (ASE) at specific cancer driver gene loci [7, 8]. ASE is the phenomenon whereby two or more gene alleles are differentially expressed with respect to one another [9]. The potential clinical consequences of ASE have been previously documented [10] including emerging evidence for the potential contribution of ASE to cancer [8, 11].

If cancer driver mutations can be transcriptionally repressed/de-repressed in an allele-specific manner, it follows that mutations in these genes may be necessary but not always sufficient for onset and progression of the disease. For example, cancer driver mutations may, to a greater or lesser extent, be repressible and thus segregating at higher than expected frequencies in populations of normal healthy individuals. In addition, regulatory modulations in the ASE of cancer driver mutations may themselves, in at least some instances, be a significant contributor to cancer onset and progression. Of particular interest, in this regard, are those genes where loss-of-function (LOF) mutations have been shown to drive cancer onset/progression. This class of cancer driver genes is commonly known as tumor suppressor genes (TSGs) because a functional wild-type allele is considered sufficient to “suppress” the cancer driver effect of LOF alleles in heterozygotes. While LOF tumor suppressor mutations are typically considered to be recessive [12], if these mutant alleles can be significantly differentially expressed relative to wild-type alleles in heterozygotes, the clinical consequences could be significant.

In this study, we first demonstrate that LOF TSG alleles are segregating in world-wide populations of normal/healthy individuals at relatively high frequencies, thereby establishing the potential importance of these genes in pre-disposing otherwise healthy individuals to cancer. To directly evaluate the possible contribution of ASE of tumor suppressor LOF alleles in cancer onset/progression, we analyzed matched sets of normal and tumor tissues isolated from 233 cancer patients representing four diverse tumor types. The results indicate that there are functionally important changes in ASE in individuals heterozygous for LOF TSG alleles associated with cancer onset/progression. While a variety of molecular mechanisms were identified as potentially contributing to changes in ASE in cancer, changes in DNA copy number and allele-specific alternative splicing possibly mediated by antisense RNA emerged as predominant factors.

## RESULTS

### Tumor suppressor mutations are abundant in human populations

The Catalogue Of Somatic Mutations In Cancer (COSMIC) is the world’s largest database of somatic mutations associated with cancer onset and progression [13]. To determine the extent to which cancer associated mutations are segregating in the general human population, the genomic locations of all coding mutations in COSMIC census genes were intersected with sequence variants identified in individuals comprising the Phase 3 release of the One Thousand Genomes Project (1KGP). The Phase 3 release catalogues all of the genetic variants present in 2504 putatively healthy individuals, representing a diversity of racial and ethnic groups randomly selected from 26 human populations around the world.

**Figure 1:**
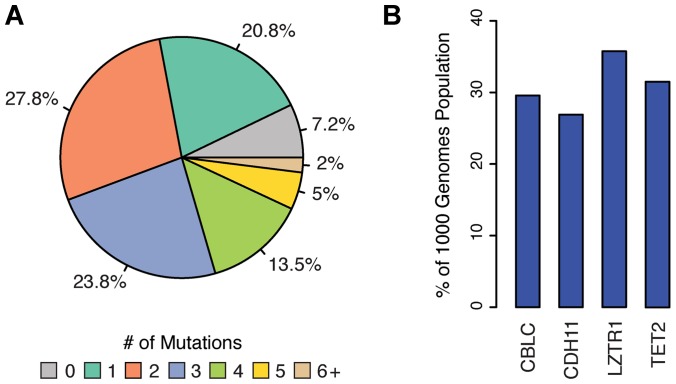
Distribution of LOF COSMIC census mutations in TSGs of the 1KGP. Cancer associated mutations were identified in the 1000 genomes population (1KGP) as detailed in the Materials and Methods. (**A**) Pie chart depicting the percent of the 1KGP containing deleterious cancer associated mutations in at least one TSG. (**B**) Four TSGs most frequently mutated (LOF) in 1KGP.

Remarkably, all individuals in the 1KGP were found to contain at least 31 homozygous and 68 heterozygous COSMIC census mutations (Supplementary Figure 1). In total, 2,296 and 3,123 COSMIC census mutations were found in oncogenes and tumor suppressor genes, respectively, in healthy individuals. However, since the functional significance of all COSMIC mutations is not yet known and the fact that gain-of-function (dominant) mutations are difficult to unambiguously identify [14], we focused our subsequent analyses on COSMIC mutations in TSGs that could be definitively classified as deleterious (*i. e*., non-sense, frame-shift, deletion mutations), along with all missense mutations predicted to be damaging by both The Sorting Intolerant from Tolerant (SIFT) [15] and Polymorphism Phenotyping v2 (PolyPhen-2) [16] algorithms. Employing this more conservative metric, 448 LOF COSMIC census mutations (28 truncating, 420 missense predicted damaging) in TSGs were identified (Supplementary Dataset 1), of which ~93% of individuals carried at least one (Figure 1A). These 448 LOF mutations mapped to 137 different TSGs in at least one individual and four of these TSGs, Cbl Proto-Oncogene C (*CBLC*), Cadherin 11 (*CDH11*), Leucine Zipper Like Transcription Regulator 1 (*LZTR1*), and Tet Methylcytosine Dioxygenase 2 (*TET2*) had LOF mutations in >25% of the population (Figure 1B). Collectively, these findings indicate that genetic variants previously characterized as “cancer driver” mutations are segregating at relatively high frequencies in populations of individuals not afflicted with the disease.

### A minority (<20%) of TSGs display genetic profiles in the cancer samples that are consistent with Knudson’s two-hit hypothesis

Given the relative abundance of TSG LOF alleles in human populations, we utilized The Cancer Genome Atlas (TCGA) database [17] to explore the possible contribution of these genes to cancer onset and/or progression by examining matched sets of cancer and normal tissues collected from 233 cancer patients representative of four diverse cancer types (breast invasive carcinoma, head and neck squamous cell carcinoma, lung adenocarcinoma, and thyroid carcinoma). According to a model first proposed by Alfred Knudson in 1971 [18], newly arising LOF TSG mutant alleles, being recessive, can be carried by normal cells with little significant negative effect. According to this model, acquisition of a second LOF mutation in the alternate wild-type allele is pre-requisite for tumor onset.

To test this hypothesis in our dataset, we genotyped all samples and identified TSGs that were heterozygous for a LOF mutation in normal tissues but that have acquired a secondary LOF mutation in the wild-type allele in the tumor samples. In total we found that only 46 of the 233 cancer patients (19.7%) were associated with acquisition of homozygosity in cancer for LOF alleles at TSG loci consistent with Knudson’s “two-hit” hypothesis. These results indicate that the vast majority of TSGs heterozygous for wild-type and LOF alleles in normal tissues remain heterozygous in tumor tissue. However, if recessive LOF alleles can be significantly overexpressed relative to the wild-type alleles in an ASE fashion, LOF TSGs may be significant contributors to cancer onset/progression even in the heterozygous state.

### The proportion of LOF mutations displaying ASE is significantly elevated in cancer tissue samples

To explore the possible contribution of ASE in matched sets of normal and cancer tissues, we employed DNA-seq data from the TCGA database to identify all heterozygous sites in the exome and subsequently leveraged complementary RNA-seq data to compare the expression of wild-type or “reference” (ref) vs LOF mutant or “alternative” (alt) alleles at those loci (Supplementary Figure 2).

**Figure 2:**
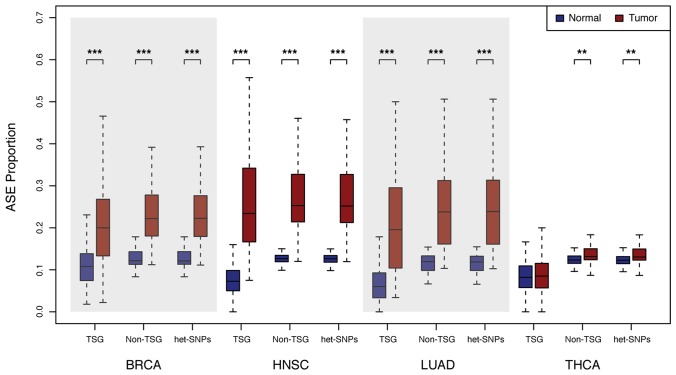
Distribution of the proportion of ASE loci. Allele counts were generated for normal and primary tumor tissue pairs for breast invasive carcinoma (BRCA), head and neck squamous cell carcinoma (HNSC), lung adenocarcinoma (LUAD), and thyroid carcinoma (THCA) via analysis of RNA-Seq as described in the Materials and Methods section. Boxplots show the distribution of the proportion of heterozygous COSMIC Census mutations in tumor suppressor genes (TSGs), all heterozygous SNPs in non-tumor suppressor genes (Non-TSG) and all heterozygous single nucleotide polymorphisms exome-wide (All het-SNPs) with significant ASE (FDR = 5%, *P* < 0.005) in normal (blue) and tumor (red) samples (^***^ *P* < 3.46 × 10−15; ^**^ *P* < 0.005).

The proportion of COSMIC census mutations in TSGs displaying ASE was found to be significantly higher in the cancer relative to normal tissues for breast invasive carcinoma, head and neck squamous cell carcinoma, and lung adenocarcinoma (*P* < 3.11 × 10^−10^) (Figure 2). Thyroid carcinoma was the only cancer type not displaying a significant difference, perhaps because these cancers are typically associated with a relatively low mutation rate [19] resulting in relatively fewer genetic alterations.

To determine if this regulatory change was limited to TSG loci, we computed ASE for all heterozygous single nucleotide polymorphisms (het-SNPs) exome-wide. We found that all genes, on average, contain a significantly higher proportion of het-SNPs displaying ASE in breast, lung, head and neck (*P* < 3.46 × 10^−15^) and thyroid (*P* < 0.005) tumors than normal samples (Figure 2). Thus, dysregulation in cancer, at least as manifest by ASE, is not limited to TSGs but extends to genes not previously identified as being implicated in tumorigenesis.

### Differences in patterns of ASE between normal and tumor tissues includes but is not limited to TSGs

**Figure 3:**
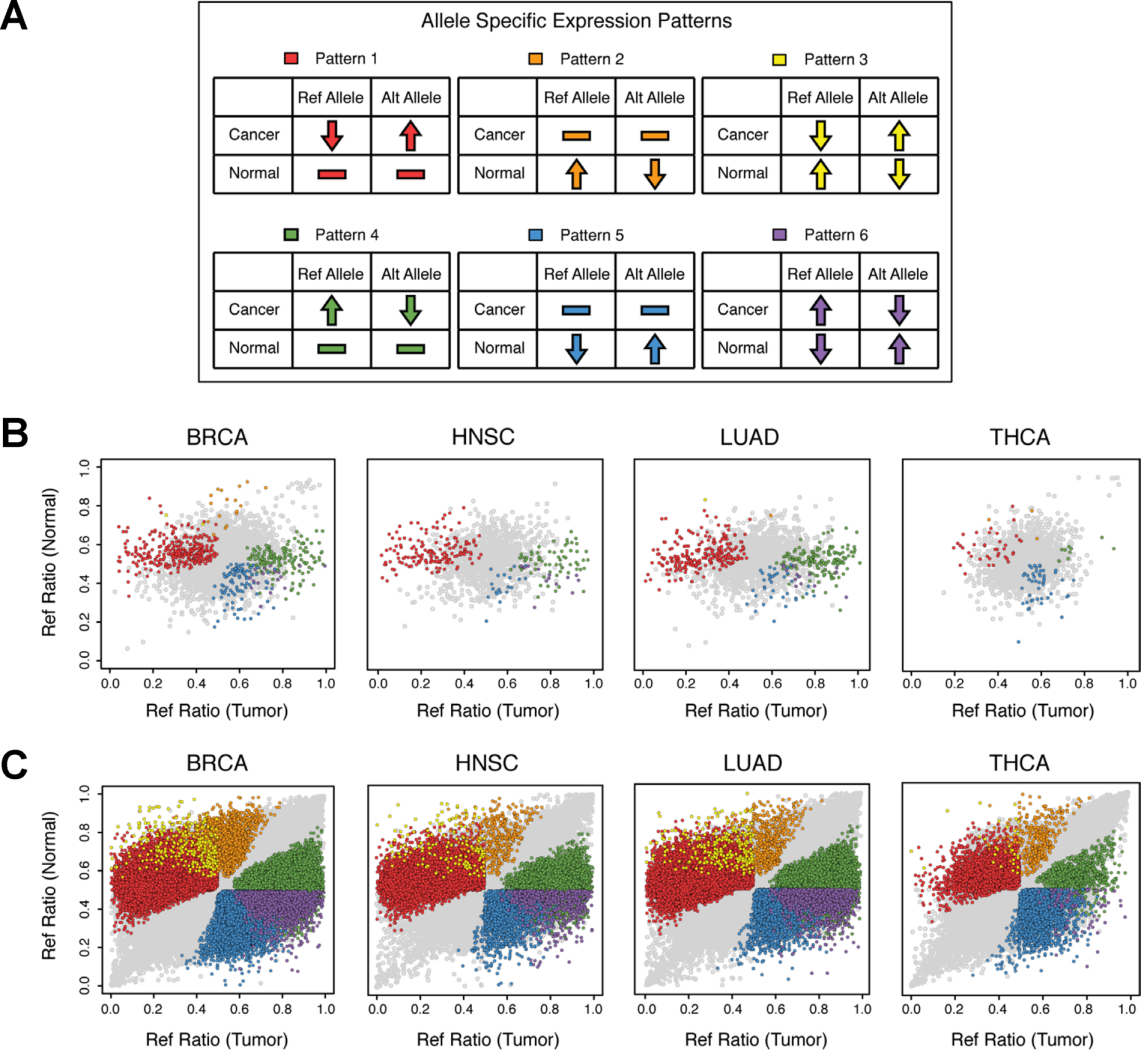
ASE SNP patterns. Allele counts were generated for normal and primary tumor tissue pairs for breast invasive carcinoma, head and neck squamous cell carcinoma, lung adenocarcinoma and thyroid carcinoma via analysis of RNA-Seq as described in the Materials and Methods section. Sites demonstrating significantly different ASE ratios (*P* < 0.05) between normal and tumor sample pairs are color coded by expression pattern as demonstrated in the top panel. (**A**) Six ASE patterns of interest were analyzed; Pattern 1: No significant difference in ASE (ref=alt) in normal tissues but significant ASE (ref<alt) in cancer tissues; Pattern 2: Significant ASE in normal tissues (ref>alt) but no significant ASE (ref=alt) in cancer tissues; Pattern 3: Significant ASE in normal sample (ref>alt) and significant ASE in tumor sample (ref<alt); Pattern 4: No significant ASE in normal tissues (ref=alt) but significant ASE in cancer tissues (ref>alt); Pattern 5: Significant ASE (ref<alt) in normal tissues but no significant ASE in cancer tissues (ref=alt); Pattern 6: Significant ASE in normal (ref<alt) and in cancer tissues (ref>alt). Significant ASE (FDR = 5%, *P* < 0.005) was determined using a binomial test within samples in order to group loci into patterns. (**B**) Reference allele ratios (ref/total) for all COSMIC Census loci in TSGs intersecting normal and tumor sample pairs, for 233 TCGA participants are shown here. (**C**) Reference allele ratios (ref/total) for all loci intersecting normal and tumor sample pairs, for 233 TCGA participants are shown here.

Changes in the relative expression of wild-type (ref) alleles vs. mutant (alt) alleles between normal and cancer tissues may manifest in one of six alternative ASE patterns (Figure 3A): Pattern 1: No significant difference in ASE (ref=alt) in normal tissues but significant ASE (ref<alt) in cancer tissues; Pattern 2: Significant ASE in normal tissues (ref>alt) but no significant ASE (ref=alt) in cancer tissues; Pattern 3: Significant ASE in normal sample (ref>alt) and significant ASE in tumor sample (ref<alt); Pattern 4: No significant ASE in normal tissues (ref=alt) but significant ASE in cancer tissues (ref>alt); Pattern 5: Significant ASE (ref<alt) in normal tissues but no significant ASE in cancer tissues (ref=alt); Pattern 6: Significant ASE in normal (ref<alt) and in cancer tissues (ref>alt). Patterns 1-3 are potentially of the most significance to cancer onset/progression because, in each case, the expression of the cancer driver LOF mutant (alt) allele is expressed at a higher level than the wild-type allele in cancer tissues.

The observed changes in ASE between matched sets of normal and cancer tissues for each of the 233 patients, grouped into their respective Patterns, is presented in Table 1. A significant percentage of mutations in TSGs were found to display various patterns of ASE (FDR = 5%, *P* < 0.005; breast 14.9%, head and neck 16.0% and lung 19.6%) with Patterns 1 and 4 being the most predominant (see also Figure 3B). Thyroid cancer again stood out as an outlier where only 4.1% of mutations in TSGs were found to display ASE with Patterns 1 (2.3%) and 5 (1.3%) being nearly equally abundant.

**Table 1:** Percent of SNPs displaying ASE in 233 TCGA patients

	Pattern	% Total SNPs	% All COSMIC	% TSG	% Oncogene	% Fusion
**BRCA**	1	7.3	7.6	7.3	7.0	8.3
	2	0.4	0.8	0.3	0.4	1.4
	3	0.1	0.2	0.0	0.0	0.3
	4	3.8	4.1	3.7	3.4	4.8
	5	2.6	2.5	2.8	2.2	2.7
	6	0.5	0.5	0.8	0.4	0.4
	No ASE	85.2	84.4	85.1	86.6	82.1
**HNSC**	1	8.7	9.7	10.5	10.1	8.3
	2	0.4	0.4	0.0	0.0	0.9
	3	0.2	0.3	0.0	0.0	0.6
	4	4.6	4.5	3.7	4.2	4.7
	5	2.1	1.7	1.3	1.2	2.0
	6	0.7	1.0	0.5	0.9	1.0
	No ASE	83.4	82.5	83.9	83.5	82.4
**LUAD**	1	9.7	10.7	9.2	9.8	12.0
	2	0.3	1.0	0.1	0.0	2.0
	3	0.2	1.0	0.0	0.0	1.9
	4	5.4	6.8	7.0	6.5	6.9
	5	1.9	2.3	2.2	1.6	2.7
	6	0.7	0.8	1.1	0.6	0.9
	No ASE	81.7	77.4	80.4	81.5	73.5
**THCA**	1	2.1	2.3	2.3	1.7	2.6
	2	0.2	0.5	0.0	0.1	1.0
	3	0.0	0.0	0.0	0.0	0.0
	4	0.5	0.8	0.5	0.4	1.1
	5	1.6	1.9	1.3	2.0	2.1
	6	0.1	0.0	0.0	0.0	0.0
	No ASE	95.5	94.5	95.9	95.8	93.2

When the analysis was extended to include all transcribed genes (“%Total SNPs” in Table 1), a similar trend was observed, where 14.8%, 16.6% and 18.3% of all het-SNPs were found to display ASE in breast, head and neck and lung cancers, respectively. Thyroid cancer was again an outlier with only 4.5% of all transcribed genes displaying ASE (Figure 3C). Collectively these results indicate that changes in ASE in cancer are widespread and not limited to TSGs.

To explore this apparent dysregulation of COSMIC census mutations in TSGs further, we aggregated our SNP ASE data to quantify ASE of the entire allele of a gene by employing the Meta-analysis Based Allele-Specific Expression Detection (MBASED) protocol [8]. We found 14.4%, 17.9%, 20.4% and 5.7% of all TSGs show ASE in breast, head and neck, lung and thyroid cancers, respectively (Supplementary Table 1). These results are consistent with the relative levels of ASE associated with individual SNPs in these cancers with Pattern 1 again emerging as a predominant pattern (9.1%, 11.1%, 13.2%, and 2.8%) (Supplementary Table 1).

One example of those TSGs displaying ASE in cancer is the Human Leukocyte Antigen A1 gene (*HLA-A*). *HLA-A* has been previously identified as a hotspot for ASE activity [20], likely due to the high genetic variability that is well-documented in the major histocompatibility complex [21]. We detected ASE in the *HLA-A* gene in 20% of patient samples including nucleotide positions not previously reported to display ASE [22].

**Figure 4:**
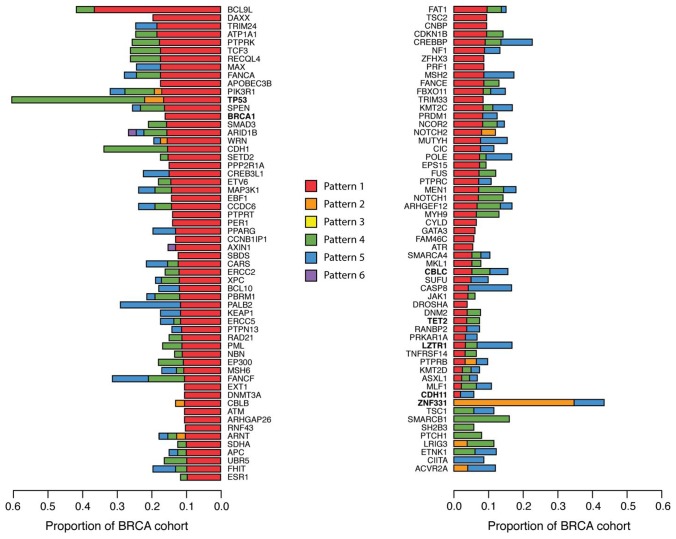
Tumor suppressor genes with ASE in breast cancer patients. Gene level ASE was computed as described in the Materials and Methods section. The proportion of breast cancer patients with ASE in 115 TSGs are shown here, colored by ASE Pattern.

Another example is Tumor Protein P53 (*TP53*) that displayed the most changes in ASE within the breast cancer patients (57.9% of all patients) displaying Pattern 4 63.6% of the time (Figure 4). Additionally, breast cancer implicated TSGs Breast cancer type 1 susceptibility protein (*BRCA1*) and Cadherin 1 (*CDH1*) were found to display changes in ASE in 15.4% and 32.4% of breast tumors, respectively, frequently displaying Pattern 1 (Figure 4). Interestingly, Zinc Finger Protein 331 (*ZNF331*) was the only TSG predominately displaying Pattern 2 (Figure 4). A previous study [23] has shown *ZNF331* to display large amounts of ASE in breast cancer, citing genomic imprinting as a possible explanation [24].

The four TSGs: *CBLC*, *CDH11, LZTR1*, and *TET2* previously shown to be most frequently mutated in 1KGP (Figure 1B) were also observed to display changes in ASE in breast cancer (Figure 4). Similar trends in the frequency of ASE Patterns among TSGs were observed in head and neck, lung, and thyroid cancers, with thyroid again sporting the least amount of ASE (Supplementary Figure 3).

### Changes in DNA allelic ratios may explain up to 35% of the observed changes in ASE between normal and cancer samples

Perhaps the most straight-forward explanation of the observed changes in ASE in cancer is that it reflects the underlying changes in allele counts on the DNA level. For example, it is known that the duplication or deletion of alleles on the DNA level can contribute to ASE in cancer [7, 25]. In addition, the polyclonal heterogeneity of most tumors can manifest as an imbalance in DNA allele counts and associated ASE changes in analyses carried out on bulk tumor samples.

**Figure 5:**
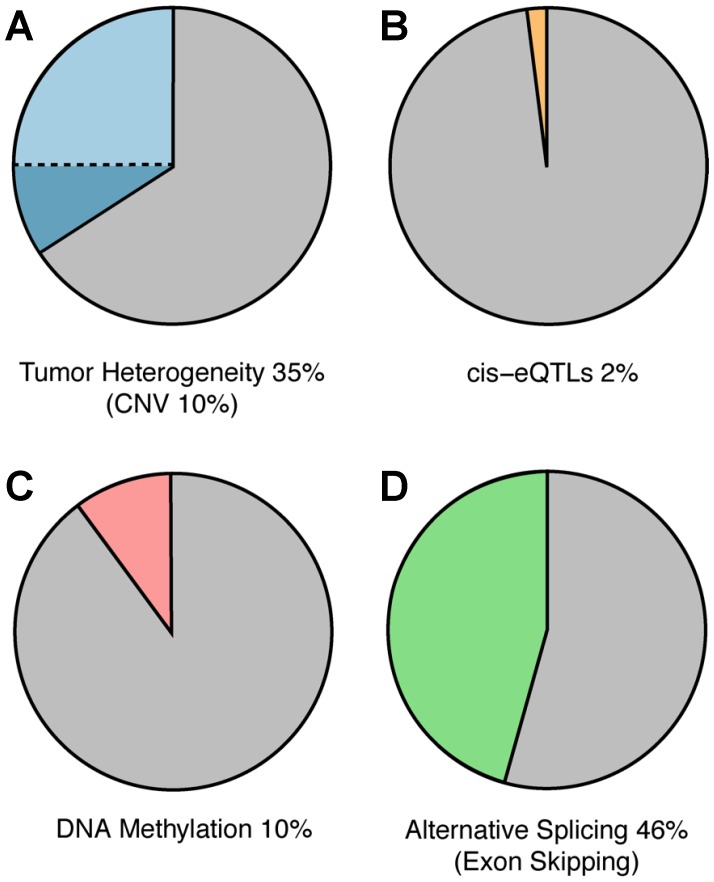
Mechanisms of ASE. Potential underlying mechanisms for ASE were explored as outlined in the Materials and Methods. Pie chart depicts amount of ASE that could be attributed to (**A**) tumor heterogeneity and copy number variation (CNV, darker blue) (**B**) *cis*-eQTLs, (**C**) DNA methylation and (**D**) exon-skipping via computational analysis.

To explore the extent to which changes in DNA copy number may be contributing to the observed ASE in the samples, we downloaded whole-exome sequencing data (WXS) for nine randomly selected patients representing each of the three cancer types displaying the highest level of ASE (breast invasive carcinoma, lung adenocarcinoma, and head & neck squamous cell carcinoma) (Supplementary Table 2). We ensured these individuals displayed ASE in COSMIC genes (Supplementary Figure 4) and that their ASE was evenly spread throughout the genome (Supplementary Figure 5). We found that, on average, 35.2% (45.7% Breast, 25.8% Head and Neck, and 20.5% Lung) of ASE genes displayed DNA allele counts that correlated with RNA allele counts (Table 2). Further investigation of these samples, however, revealed that only 10% of these genes displayed copy number duplications potentially accounting for their ASE (Figure 5A). Collectively these findings indicate that while, on average, a large fraction of the observed changes in ASE may be accounted for by corresponding changes in DNA allele counts, many instances of ASE in the cancer samples are likely attributable to allele-specific changes in gene regulation.

**Table 2:** ASE patterns potentially explained by DNA counts

Patient	ASE SNPs explained by DNA counts	Total ASE SNPs	Percentage of ASE correlated
Breast 1	526	1336	39.4
Breast 2	719	1411	51.0
Breast 3	177	367	48.2
Head & Neck 1	275	930	29.6
Head & Neck 2	179	674	26.6
Head & Neck 3	217	993	21.9
Lung 1	56	233	24.0
Lung 2	47	155	30.3
Lung 3	11	167	6.6
**Total**	**2207**	**6266**	**35.2**

### Allele-specific cis-regulatory variation may account for a relatively small fraction of observed changes in ASE between normal and cancer samples

Allele-specific regulatory changes in gene expression could be explained by sequence variation mapping to *cis*-regulatory regions located up- or down-stream of affected genes [26–28]. To explore the extent to which allele-specific *cis*-regulatory variation may account for ASE in cancer, we identified expression-quantitative trait loci (eQTLs) present in six of the nine patients’ normal and tumor samples using the Genotype-Tissue Expression Project’s (GTEx) single tissue *cis*-eQTL data available for breast and lung tissue [29]. eQTLs are regions of the genome containing DNA sequence variants previously established to regulate gene expression levels [30]. Genes previously established to be regulated by at least one eQTL are classified as eGenes [31].

Genes displaying ASE in our study were found to be significantly enriched for eGenes relative to genes not displaying ASE (*P* = 0.018) (Supplementary Figure 6). This finding was pronounced for breast (*P* = 2.56 × 10^−6^) and lung cancer (*P* = 6.22 × 10^−4^) patients (Supplementary Figure 6). However, collectively only 24% of genes displaying ASE in our dataset are eGenes and just 3% of ASE eQTLs are ASE-specific. Moreover, we found that the expression slope of an eQTL is not often correlated with the allelic expression of a gene (1.8%; Figure 5B; Supplementary Table 3). For example, consider the heterozygous eQTL variant (rs10654) mapping to the 3′ UTR of the *NUP54* gene in both normal and tumor samples of breast cancer patient 2 (TCGA-BH-A0BW). Despite being heterozygous, this eQTL is not associated with ASE in the normal tissue but is associated with ASE in cancer tissue where the alternative haplotype is overexpressed and in phase with the highly expressed alternative eQTL allele (Supplementary Figure 7A).

We also pursued the eQTLs differing in genotype between normal and tumor samples for specific evidence of *cis*-regulation. While infrequent, we did find several notable cases where eQTL genotypes correlated with ASE. Shown in Supplementary Figure 7B, is a model for how *cis*-eQTLs may be responsible for the intragenic ASE we observed. In this particular example, three separate eQTLs within a 50bp region (rs34176173, rs12085114, rs34016668) are found ~4.8k base pairs from the 3′ UTR of the gene *NME7* in breast invasive carcinoma patient 3 (TCGA-BH-A0DT). The eQTL is homozygous for the ref allele in the normal sample that does not show ASE, and heterozygous in the tumor sample. The eQTL alternative allele that is associated with high expression of NME7 is present on the alt haplotype being overexpressed. Further, all three eQTLs are in linkage disequilibrium with the ASE SNP (r > 0.42) suggesting they segregate together. We found four additional cases where *cis*-eQTLs could account for ASE but none of these were associated with COSMIC census genes.

Collectively, the above findings indicate that while allele-specific *cis*-regulatory variation may account for some instances of ASE, it alone does not explain the vast majority (>75%) of instances of ASE in our dataset.

### Changes in methylation do not appear to be a major contributor to the observed changes in ASE between normal and cancer samples

Another possibility is that ASE is regulated epigenetically. For example, it has been previously suggested that epigenetic inactivation of one of the two alleles could result in ASE [32]. Epigenetic effects across chromosomes are often regionally associated with CpG repeats or “CpG islands” [33]. To determine if genes displaying ASE in our dataset display evidence of regional chromosomal clustering, we visualized the genomic locations of ASE for nine patients on a genome ideogram (Supplementary Figure 5). The results provide no evidence for regional chromosomal clustering indicative of regional epigenetic effects.

To further search for evidence of epigenetic involvement in ASE in our dataset, we analyzed global DNA methylation in normal and cancer tissues since this is a common mechanism by which gene transcription can be repressed epigenetically [34, 35]. Methylation data were downloaded from TCGA for seven of the nine patients described above and used to compare genes that had a significant change in methylation with genes showing a significant change in ASE. We found that only 10.2% of genes displaying ASE also displayed significant differences (>1.3-fold) in methylation between normal and tumor tissues (Figure 5C; Supplementary Table 3). Although these results indicate that changes in methylation are not likely to be playing a significant role in the ASE detected in patient samples, the analysis cannot be considered definitive because the methylation data provided by TCGA are not allele specific.

### A significant fraction of changes in ASE between normal and cancer may be a reflection of underlying alternative-splicing events

A recent study has implicated allele-specific alternative splicing as a potentially significant factor in ASE [36]. For example, consider a scenario where an allele-specific exon-skipping event occurs more frequently in a cancer tissue than normal (Supplementary Figure 8). This would result in a negligible difference in the level of transcripts containing the wild-type (ref) and LOF mutant (alt) allele in normal but significantly fewer transcripts containing the wild-type allele (“T allele”, in Supplementary Figure 8) in cancer.

To explore the possibility that allele-specific alternative splicing may be contributing to the observed ASE in patient samples, we leverage previously computed isoform counts for TCGA patient data [37]. Specifically, we sought to determine if there is a significant increase in exon skipping in genes displaying ASE. The results indicate that 46% of SNPs displaying changes in ASE between normal and cancer correlate with an increased frequency of exon-skipping events (i. e., ≥1.5-fold increase in expression of reads consistent with exon-skipping events) (Table 3; Figure 5D).

**Table 3:** ASE SNPs with differentially expressed exon-skipping events and enrichment of antisense RNA

ASE Pattern	Total ASE SNPs	ASE SNPs w/ 1.5× ISO1^a^	Percentage of ASE correlated	ASE SNPs w/ 1.5× ISO1 and 1.5× AS^b^	Percentage of ISO1 correlated w/ antisense
1	591	278	47.0	191	32.3
2	13	5	38.5	1	7.7
3	4	4	100.0	4	100.0
4	500	217	43.4	155	31.0
5	59	18	30.5	7	11.9
6	71	51	71.8	51	71.8
**Total**	1238	573	**46.3**	409	**33.0**

^a^Reads supporting ISO1 (isoform 1 genelet) are split-reads spanning the two flanking exons adjacent to the skipped exon

^b^RNA reads with predicted antisense orientation.

While these results suggest that allele-specific alternative splicing may be a significant contributor to ASE, it does not provide a mechanism as to how two variant alleles from the same gene may be alternatively spliced. One possibility is that the point mutations or indels that distinguish mutant LOF (alt) alleles from wild-type (ref) alleles map to consensus splice sites or other *cis*-regulatory locations known to be involved in the splicing process [38]. However, of the 100,852 SNPs associated with changes in ASE between normal and tumor, only 1.4% (1,418/100,852) map to consensus splice sites (716 in acceptor G, 702 in donor AG) (Supplementary Dataset 2).

A second possible mechanism that may explain how two variant alleles from the same gene may be alternatively spliced emerges from previous studies showing that splicing events can be experimentally induced *in vivo* by exposing primary transcripts to even small fragments of antisense RNAs that pair with known splice sites in the primary transcript [39, 40]. We reasoned that if such allele-specific antisense RNAs are being differentially produced in normal and cancer tissues, it may explain observed differences in allele-specific splicing and consequent differences in ASE.

**Figure 6:**
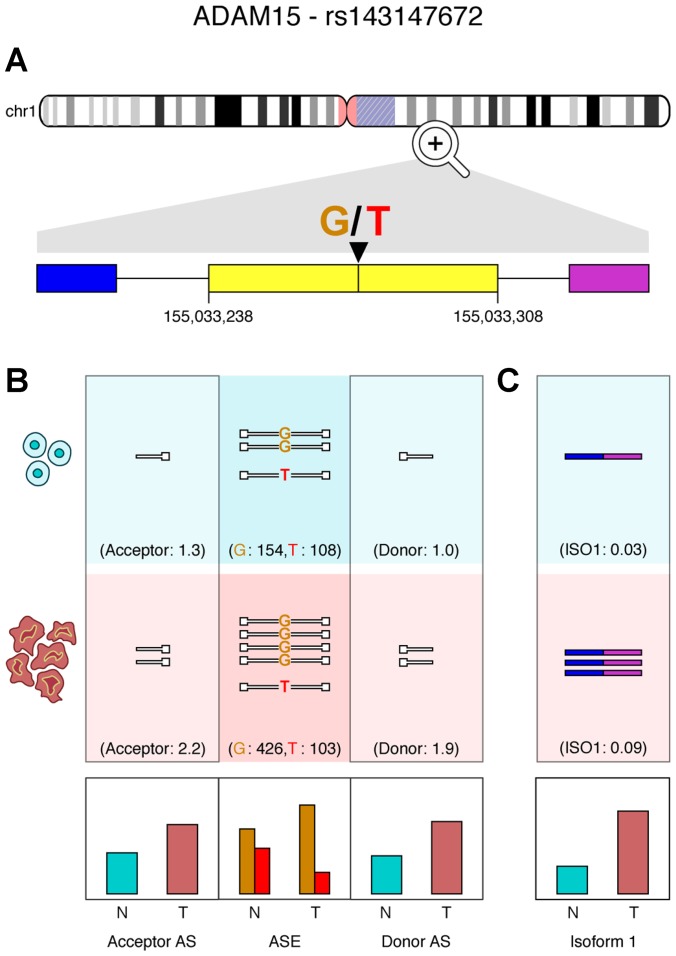
ADAM15 exon skipping correlates with ASE in a breast adenocarcinoma patient. (**A**) An exon-skipping event in exon 19 of ADAM15 in a breast cancer patient (TCGA-BH-A0DT). (**B**) Antisense reads ( 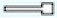) mapping to donor and acceptor sites are quantified, alongside the ASE locus within the exon (N = normal; T = tumor; AS = antisense RNA). (**C**) Quantification of reads supporting the isoform missing exon 19. Relative expression plots are shown for antisense RNA, ASE and isoforms below.

To test this hypothesis, we estimated the levels of antisense RNAs mapping to splice sites adjacent to allele-specific alternative-splice events. The results presented in Table 3 demonstrate a notable increase in levels of antisense RNA in genes displaying allele-specific alternative-splice events associated with ASE. For example, Figure 6A depicts a case where the *ADAM15* gene displays ASE in breast cancer patient 3 (TCGA-BH-A0DT). The ADAM15 protein is known to display tumor suppressive activities when it is released as an exosomal component [41], and abnormal expression and dysregulation of alternative splicing in *ADAM15* has been previously associated with breast cancer [42]. Previous studies have also shown that four ADAM15 isoforms varying by the sequence of the cytoplasmic domains, display variable effects *in vitro*. The shortest isoform, ADAM-15D, arises due to loss of exons 19 to 21 causing a reading frame shift in exons 22 and 23 when compared with the other three isoforms. The variant lacks proline-rich modules and has a distinct sequence of 37 amino acids. As shown in Figure 6B, we observe an increase in antisense RNA mapping to acceptor (1.7×) and donor (1.8×) sites in this patient’s tumor. We have identified an exon-skipping event (exon 19), consistent with the ADAM-15D isoform. The increase in antisense RNA correlates with this isoform’s expression, which is substantially higher (3.8×) in the patient’s tumor sample when compared to normal and could explain ASE at this locus (Figure 6C).

Another example is illustrated in Supplementary Figure 9A, where the Lysyl Oxidase Like 2 (*LOXL2*) gene displays ASE at the *rs1051146* locus in breast cancer patient 1 (TCGA-BH-A0B3), which overlaps with an exon-skipping event. *LOXL2* has accumulated numerous reports that document its role in cancer formation and proliferation of breast cancer [43, 44]. Further, research has shown that a short isoform of LOXL2 missing exon 13 can regulate cancer cell migration and invasion through a dissimilar mechanism compared to its canonical form [45]. Here, we observe more antisense RNA mapping to acceptor (8.7×) and donor (9.2×) sites (Supplementary Figure 9B) and increased skipping of exon 6 (9.9×) (Supplementary Figure 9C) in breast cancer patient 1’s tumor sample, both correlated with an increase in ASE.

Tenascin C (*TNC*) is a gene belonging to a family of extracellular matrix (ECM) glycoproteins that is known to be overexpressed in cancer cells. Studies have shown that remodeling of ECM in cancer can affect cellular interaction as ECM influences behavior of the cells [46, 47]. One specific study has shown that a TNC isoform containing exons 14 and 16 but not 15 is upregulated in breast cancer, which leads to increased cell invasion and proliferation [48]. In breast cancer patient 3, *TNC* displays changes in ASE at *rs17819466* inside exon 15 (Supplementary Figure 10A). Antisense RNA mapping to acceptor (2.1×) and donor (2.6×) sites are elevated in the tumor sample (Supplementary Figure 10B), as are split-reads spanning exons 14 and 16 (5.5×) (Supplementary Figure 10B).

Collectively, these results suggest that antisense RNA mediated alternative splicing may be a significant factor in accounting for our observed changes in ASE between normal and cancer samples.

## DISCUSSION

Cancer is a complex disease not only from the perspective of the number and diversity of genes involved but also because of the existence of extensive regulatory variation controlling the expression of these genes. One manifestation of these regulatory controls is allele-specific expression (ASE) at specific cancer driver gene loci [7]. If cancer driver mutations can be transcriptionally repressed/de-repressed in an allele-specific manner, they may be segregating at higher than expected frequencies in populations of normal healthy individuals. In an initial effort to explore this possibility, we conducted a computational analysis of functionally significant cancer driver mutations in a sampling of normal healthy human populations across the world (2.5 thousand genomes comprising the 1000 Genome Project (1KGP) [49]). While relatively few confirmed dominant oncogene mutations were found to be segregating in these populations, 93% of healthy individuals sampled were found to carry functionally significant loss-of-function (LOF) cancer driver mutations at one or more tumor suppressor gene loci (21% of individuals carry 1 mutant allele; 28% carry 2, 24% carry 3, 13% carry 4, 5% carry 5, 2% carry >6). This encompassed 448 LOF mutations (averaging 3.2 LOF mutations per TSG), 420 of which are computationally predicted to be deleterious without experimental validation. In contrast, we found that the frequency of such LOF mutations is higher in random samplings of non-TSGs (Supplementary Table 4) as well as in TSGs from normal tissues in TCGA patients (Supplementary Table 5) consistent with the idea of negative selection against LOF somatic mutations that affect TSGs.

While the frequencies of LOF TSGs we detected are higher than what has been typically reported for specific TSGs [50, 51], they are not unprecedented. For example, among the most intensively studied TSGs is the *RB1* gene that is associated with inherited childhood retinoblastoma [18]. Although the frequency of individuals heterozygous for LOF RB1 alleles (“carriers”) in human populations is generally reported to be ≤ 5% [51], considerable variability exists among ethnic groups/populations. For example, in a study of select Asian populations, the frequency of carriers of LOF RB1 alleles was reported to be as high as 34% in specific ethnic populations [52].

The “two-hit” hypothesis proposes that individuals heterozygous for a LOF tumor suppressor allele will not typically develop cancer unless an additional LOF mutation occurs in the gene’s functional partner allele [18]. While the two-hit hypothesis has been successfully employed to account for many instances of inherited cancers associated with tumor suppressor genes [53, 54], a number of examples have been identified in recent years that are inconsistent with Knudson’s “two-hit” hypothesis [55–57]. For example, it is now known that not all children afflicted with retinoblastoma are homozygous for the LOF RB1 allele [58, 59] and this condition has, in several cases, been associated with aberrant expression of unlinked regulatory genes [60]. While evaluating the “two-hit” model in our dataset, we found that < 20% of patients acquired a second LOF mutation in cancer tissues as predicted by the “two-hit” model. This finding is consistent with a growing body of evidence that the mechanisms underlying the contribution of TSGs to cancer onset and progression are often more complex than originally envisioned [61, 62].

A primary goal of our study was to evaluate the potential significance of changes in ASE of TSGs between normal and cancer, and to explore the molecular mechanisms that may underly this process. While searching the TCGA database for evidence of ASE we found that COSMIC census mutations in TSGs display significantly (*P* < 3.11 × 10^−10^) more ASE in tumors compared to matched normal tissues in breast, head and neck, and lung cancers. Our finding that this change is not limited to COSMIC genes but extends to genes not previously associated with cancer, implies a general loss of regulatory control in cancer. Evidence for such a global loss in regulatory control in cancer has been previously reported [63, 64]. This difference in ASE between tumors and matched normal tissues was substantially less and not significant within TSGs in thyroid carcinoma. Further research will be required to unequivocally determine the basis for this discrepancy. However, one possibility is that thyroid cancer’s inherently low mutation rate [19] allows for its transcriptional regulation to remain more intact.

Of the six possible Patterns of change in ASE between normal and cancer tissues, we found that Pattern 1 (*i. e*., no ASE in normal tissue but expression of mutant allele (alt) > expression of wild-type (ref) allele in cancer tissue) was one of the most commonly observed Patterns across cancer types. This finding is consistent with the hypothesis that LOF TSG alleles may be contributing significantly to cancer onset/progression even in the heterozygous state.

One possible explanation of the observed changes in ASE patterns between normal and cancer tissue is that it is structural in nature, *i. e*., the consequence of differences in allele counts attributable to, for example, loss of heterozygosity (LOH) or the polyclonal heterogeneity characteristic of most tumors [25]. To test this possibility, we compared RNA allele counts with DNA allele counts in the same patient samples. We found that on average, 35% of genes displaying ASE had DNA allele counts that correlated with RNA allele counts. These results are consistent with prior findings indicating that a significant fraction of ASE can be accounted for by underlying differences in DNA allelic content [25]. It should be noted that structural changes introduced by somatic mutations that create premature termination codons and/or induce nonsense-mediated RNA decay could also be a contributing factor [65]. Nevertheless, collectively our results indicate that, at least with respect to our patient samples, differences in ASE between normal and cancer tissues is not merely structurally based but likely attributable to allele-specific differences in gene expression.

Another mechanism of regulatory change of emerging significance in cancer is epigenetics (83). In a preliminary effort to explore the possible contribution of epigenetics to global changes in patterns of ASE, we analyzed methylation data for patient samples from TCGA. We found that only 10% of genes displaying ASE also displayed significant differences (>1.3-fold) in methylation between the normal and tumor tissue samples. Because changes in methylation are generally considered to be a reliable indicator of epigenetic-associated changes in gene expression [66], our results suggest that changes in methylation may not be playing a predominant role in the regulation of ASE in our patient samples.

Allele-specific differences in gene expression may also be attributable to variant *cis*-regulatory sequences located up- or down-stream from the respective alleles’ coding regions. Such *cis*-regulatory variation is commonplace and is often identified by utilizing QTL mapping methodologies [67]. We employed the Genotype-Tissue Expression Project’s (GTEx) single tissue *cis*-eQTL database to explore the extent to which allele-specific *cis*-regulatory variation may account for ASE in patient samples. We found that only 24% of genes displaying ASE are eGenes, more of which explain ASE in normal (38%) than tumor (21.8%) samples. Moreover, only 1.8% of ASE haplotypes were found to be in phase with an eQTL indicating that *cis*-regulatory variation is not a likely explanation of the majority of instances of ASE in our dataset.

Having failed to identify a mechanism of transcriptional level regulation that could explain the majority of observed instances of ASE in our dataset, we turned our attention to the potential influence of post-transcriptional regulation on ASE. One post-transcriptional mechanism of growing prominence in cancer biology is alternative splicing [68]. The primary RNA products of genes are processed at the post-transcriptional level by alternative RNA splicing resulting in multiple RNA isoforms per gene. If alternate RNA isoforms are generated on an allele-specific basis (allele-specific alternative splicing), it could manifest itself as differences in ASE. To explore the possibility that allele-specific alternative splicing could be contributing to changing patterns of ASE in cancer, we examined isoform counts associated with our TCGA patient data [37]. We found that almost half (46%) of SNPs displaying ASE in patient samples were indeed associated with exon skipping. However, further studies that employ a more granular isoform-specific quantification around ASE loci will be needed to fully understand the workings of an allele-specific alternative splicing mechanism.

While the potential functional significance of alternative splicing in cancer has been long noted [69], the mechanisms underlying the phenomenon remain poorly understood. Because the genes displaying changes in ASE are not associated with *cis*-regulatory mutations in splice acceptor/donor sites, we focused our attention on possible *trans*-regulatory mechanisms. One possibility is that one or more of the regulatory proteins or RNAs associated with the spliceosome could be mutated or otherwise dysregulated in cancer resulting in aberrant splicing patterns [70]. However, the fact that our observed allele-specific alternative splicing was limited to only a subset of genes suggested that the underlying mechanism was of a more targeted nature.

One possibility was suggested from previous studies showing that splicing events can be experimentally induced *in vivo* by exposing primary transcripts to antisense RNAs that pair with known splice sites in the primary transcript [71, 72]. Indeed, there is growing evidence that *de novo* expression of antisense RNAs may play a significant role in the induction of alternate-splice variants [73] and that this may be a significant factor in cancer onset/progression. Our results are generally consistent with this hypothesis and suggest that allele-specific alternative splicing, possibly mediated by changes in the expression of antisense RNAs, may play a significant role in the induction of changes in ASE patterns in cancer. Further studies inducing ASE *in vitro* via use of antisense oligonucleotides will be needed to validate this hypothesis.

## MATERIALS AND METHODS

### Cancer associated mutation identification in 1000 genomes population

Using the BEDTools program [74], the genomic locations of all coding mutations in COSMIC census genes (v82) [75] were intersected with a VCF file containing all sequence variants called from the 2,504 individuals of the Phase 3 release of the 1000 Genomes Project (1KGP) [49]. The distribution of these cancer associated mutations was determined for all intersecting mutations including the subset of deleterious mutations. Variant effects were annotated using Variant Effect Predictor (VEP) using the Ensembl 91 release [76]. Mutations were considered to be deleterious if they were non-sense, frameshift, splice acceptor/donor mutations, or whole gene deletion mutations. Missense mutations predicted deleterious by both SIFT [15] and Polyphen2 [16] were also scored as deleterious mutations. Moreover, we removed any mutation that had been labeled as benign or likely benign by ClinVar [77].

### Genotyping and variant calling with WXS and variant annotation

Genotyping was implemented from WXS. SAMtools mpileup output was fed to VarScan’s mpileup2snp function in order to call variants [78]. Only reads with mapping quality > 14 were counted. Further, to call a variant, a position must have met a minimum read depth of 8, minimum allelic depth of 2 and variant allele frequency threshold of 0.2. The default *p*-value of 0.01 was used for calling variants. Variants were annotated using VEP with the same criteria mentioned as above.

### Allele specific expression (ASE) analysis

#### Counting allele-specific reads

Indexed RNA-Seq BAM files along with filtered heterozygous variants were passed to GATK’s ASEReadCounter tool [79]. At this step, only reads with minimum mapping quality and base quality scores of 20 and 30, respectively, were counted. Also, minimum depths of 20 reads per site and four reads per allele were applied. With the aim of inferring biological significance, resulting allele counts were annotated with rsid using Kaviar. Subsequently, gene names associated with particular SNPs were fetched from dbSNP using EDirect [80]. The fraction of reads containing the reference allele over the total number of reads at a given position (Ref Ratio) was calculated for all heterozygous SNPs. Custom scripts were written to perform allele specific expression analysis.

#### Accounting for mapping bias

When mapping RNA-seq reads to the reference genome, reads overlapping a SNP that contain the alternative allele tend to map less frequently than those reads containing the reference allele. This allelic mapping bias has been well documented and presents challenges in ASE analysis [81]. Degner *et al*. demonstrated that the reliability in ASE estimation is greatly dependent on the capability to control for reference mapping bias [82]. To limit this bias, we first removed sites known to be susceptible to mapping bias. We did so by removing all sites with 50bp mapability < 1 based on the UCSC mapability track [83]. To correct for any residual bias, we calculated the genome-wide allelic ratios for all nucleotide pairs and used them in place of 0.5 as the expected allelic ratio in the binomial test (Supplementary Figure 11) as previously done by Lappalainen *et al* [84].

#### ASE-analysis

Using the allele counts for every heterozygous position that met our filtering requirements, we performed a binomial test to identify whether the ratio of reference and alternative read counts differed significantly from the corresponding expected proportion between those alleles. Expected ratios were inflated slightly from 0.5 based on the observed allele counts within our population as described in the previous section. We classified a site as an ASE SNP if its binomial *p*-value was less than 0.005 and corrected for a false discovery rate (FDR) of 5%. Gene level ASE was determined by aggregating ASE information from all heterozygous SNPs within a gene as outlined by the MBASED protocol [8]; ASE genes were classified with a major allele frequency (MAF) greater than 0.7 and *p*-value less than 0.05 (FDR 5%). To label significant ASE genes with Patterns we pseudo-phased them by creating a major haplotype consisting of the alleles with higher RNA read counts. If a haplotype contained more reference SNPs it was labelled as the reference haplotype and *vice versa* for the alternative. If the number of reference and alternative SNPs on each haplotype were the same, the haplotype was labelled as ambiguous.

Differences in ASE between normal and cancer tissue groups, were evaluated by comparing the distributions of the proportion of SNPs with ASE within each collection. The statistical significance levels of the observed difference in ASE between normal and tumor tissues for both COSMIC census mutations and all heterozygous SNPs were evaluated by comparing these distributions using the non-parametric Mann-Whitney *U* test.

When comparing SNPs intersecting paired normal and tumor samples, we applied a combined binomial-Fisher test to determine if ASE patterns were significant. Three ASE patterns of interest were analyzed; Pattern 1: No significant difference in ASE (ref=alt) in normal tissues but significant ASE (ref<alt) in cancer tissues; Pattern 2: Significant ASE in normal tissues (ref>alt) but no significant ASE (ref=alt) in cancer tissues; Pattern 3: Significant ASE in normal sample (ref>alt) and significant ASE in tumor sample (ref<alt); Pattern 4: No significant ASE in normal tissues (ref=alt) but significant ASE in cancer tissues (ref>alt); Pattern 5: Significant ASE (ref<alt) in normal tissues but no significant ASE in cancer tissues (ref=alt); Pattern 6: Significant ASE in normal (ref<alt) and in cancer tissues (ref>alt). All Patterns are visualized in Figure 3A. Significant ASE (FDR = 5%, *P* < 0.005) was determined using a binomial test within samples and subsequently a Fisher’s exact test (*P* < 0.05) when comparing two samples. Both tests were applied to increase stringency and validity of results.

The analyses used to determine mechanisms of ASE are outlined in detail in the Supplementary File: Materials and Methods.

### Second site loss-of-function mutations

Filtered heterozygous sites in tumor suppressor genes (TSGs) of all 233 patients in normal and tumor samples were phased using SHAPEIT [85]. Loss-of-function mutations were defined as stop gained, frameshift, splice acceptor/donor, start lost and stop lost mutations. Deleterious missense mutations predicted to be damaging/deleterious by SIFT [15] and Polyphen2 [16] were also considered loss of function in TSGs. Patients with a secondary site loss-of-function mutation were defined as having a heterozygous mutation in the normal sample and either: 1) the same mutation homozygous in the tumor sample, 2) a new loss of function mutation on the opposite allele in the tumor sample (*i. e.* compound heterozygote), or 3) a DNA segment with loss of allele at the locus in the tumor sample. Segments of DNA with loss of allele were identified using FACETS [86].

Detailed materials and methods are included in the Supplementary File.

## CONCLUSIONS

We have shown that LOF TSGs are segregating in human populations at significant frequencies suggesting that many otherwise healthy individuals are at elevated risk of developing cancer. Changes in ASE between normal and cancer tissues indicates that LOF TSG alleles may contribute to cancer onset/progression even when heterozygous with wild-type functional alleles. While a variety of molecular mechanisms were identified as potentially contributing to changes in ASE between normal and cancer, differences in DNA counts and allele-specific alternative splicing emerged as predominant factors.

## SUPPLEMENTARY MATERIALS











## Abbreviations

TSGs: Tumor suppressor genes
1KGP: 1000 Genomes Project
ASE: Allele-specific expression
LOF: Loss of function
COSMIC: Catalogue of somatic mutations in cancer
TCGA: The Cancer Genome Atlas
