# Supplementary material for: Tumor suppressor genes and allele-specific expression: mechanisms and significance
Source: Oncotarget. 2020 Jan 28;11(4):462–79. doi: 10.18632/oncotarget.27468 (PMC6996918; doi:10.18632/oncotarget.27468)
Supplement: Supplementary file 1 [file oncotarget-11-462-s001.pdf]

Tumor suppressor genes and allele-specific expression: mechanisms and significance

SUPPLEMENTARY MATERIALS

MATERIALS AND METHODS

Sequencing data acquisition

Whole exome sequencing (WXS) and transcriptome sequencing (RNA-Seq) data for matched sets of normal and primary tumor samples collected from 233 cancer patients were downloaded from The Cancer Genome Atlas (TCGA) via Genomic Data Commons (GDC) (89 Breast Cancer (BRCA) patients, 52 Lung Adenocarcinoma (LUAD) patients, 39 Head and Neck Squamous Cell Carcinoma (HNCSC) patients and 28 Thyroid Cancer (THCA) patients; Supplementary Dataset 3). These four cancer types were selected as they contained the most available DNA and RNA sequencing data for matched normal and primary tumor samples. The data consisted of paired-end reads generated from Illumina platforms. As per the harmonization pipeline of GDC all WXS samples were aligned to GRCh38 reference genome [1], indels were locally realigned and base quality scores were recalibrated. RNA-Seq samples were also aligned to GRCh38 by the GDC. Metadata for all sequencing files are summarized in Supplementary Dataset 4. Autosomes were subsequently extracted for downstream analysis using SAMtools [2]" . To investigate the mechanisms of ASE Whole Genome Sequencing data (WGS) for nine of these patients (three breast invasive carcinoma, three head and neck squamous cell carcinoma and three lung adenocarcinoma) were downloaded. The BAM files for WGS were checked for quality using FASTQC, lifted over to GRCh38 and local realignment of indels was performed using GATK [3].

Cis expression quantitative trait loci (cis-eQTL) aAnalysis

In order to investigate the possible contribution of upstream/downstream regulatory variation to ASE, *cis*-eQTL detection was performed. Variants were called, using SAMtools mpileup and bcftools [2], on the genomic sequence ± 1mb from the transcription start site (TSS) of each gene. Mono-allelic sites were phased using SHAPEIT [4] with the 1KGP reference panel of haplotypes for each participant's super population. Indels and multi-allelic sites were phased using HapCUT [5]. Subsequently, the outputs of the two tools were merged to complete the phasing step. Correlated *cis*-eQTL-gene pairs, for lung and breast tissues, were downloaded from The Genotype Tissue Expression Project's (GTEx) single tissue *cis*-eQTL data [6]. The eQTL-gene pairs were intersected with variants of the breast and lung cancer TCGA participant samples. We then created a list of all possible eQTL SNP – ASE SNP pairs, by pairing eQTL variants with all ASE SNPs in the corresponding eGene. We tested to see if there was a difference in the enrichment of eQTLs in ASE genes versus non-ASE genes using the Fisher's exact test, across all individual samples. Resulting eQTL SNP – ASE SNP pairs were tested for linkage disequilibrium (LD) using PLINK [7] and the appropriate super population from 1KGP (EUR or AFR) based on the patients' reported race. Pairwise correlations (r) for all SNP pairs were computed. Finally, VEP [8] was used to find eQTLs in regulatory regions and genomic regions of interesting cases were visualized using the AllelicImbalance R package [9].

Tumor heterogeneity and allele specific copy number variation

To explore the possible contribution of DNA copy number variation to ASE, DNA read counts and allelic depths were generated for heterozygous sites in matched normal and tumor samples using the snp-pileup utility provided in the FACETS package [10]. A binomial test with an expected ratio of 0.5 and an FDR of 5% were used to select sites where the allelic depth of the reference and alternative allele were significantly different. These sites were overlapped with ASE SNPs to see how much of the observed ASE could be attributed to the heterogenous nature of tumor tissues and copy number variations.

Segments with non-diploid copy number variations or evidence of loss of allele were identified using FACETS with a c value of 100. To calculate the amount of ASE associated with copy number variation, ASE SNPs were overlapped with non-diploid copy number variation segments to see if the copy number change supported the allelic imbalance observed in the RNA-Seq reads.

Methylation

To investigate the possible contribution of methylation to ASE, Illumina Infinium HumanMethylation450 (HM450) and Human Methylation 27 (HM27) Array data were downloaded from TCGA for six patients (TCGA-50-5932, TCGA-BH-A0B3, TCGA-BH-A0BW, TCGA-BH-A0DT, TCGA-CV-6959, TCGA-CV-7255) for tumor and normal samples. Methylation intensity was quantified by a beta-value calculated as the ratio of the methylated probe intensity and the sum of the methylated and unmethylated probes. Fold changes of the beta-values were calculated between the tumor and normal samples; a fold change of ≥ 1.33was considered significant. To estimate how many changes in ASE between tumor and normal samples could be accounted for by epigenetic changes in methylation we overlapped genes that had a significant change in methylation with genes showing a change in ASE.

Alternative splicing

To explore the possible contribution of post-transcriptional alternative splicing to ASE, a compressed alternative splicing dataset from a study [11] containing over 8,000 patients across 32 cancer types on TCGA was downloaded from GDC. Exon-skipping event data for 233 patients in this ASE study were matched and pulled from the dataset for further analyses.

In order to compare the expression of a genelet that provides evidence for an exon-skipping event that could be contributing to observed ASE in matched normal and tumor samples, the genomic coordinates of ASE SNPs were first intersected with the start and stop positions of all confirmed exon-skipping events using BEDTools. Kahles *et al*. [11] define ISO1 and ISO2 genelets as isoforms with shorter and longer lengths, respectively. For exon-skipping events, the ISO1 genelet refers to the boundary connecting the exons adjacent to the one being skipped. For the filtered list of exon-skipping events that intersect with an ASE SNP, the difference in the number of multi-exon spanning reads (ISO1) between normal and its paired tumor sample were inspected for each of the ASE patterns. The isoform counts were normalized to counts per million (CPM) using the total number of reads to account for sequencing depth. For ASE SNPs with Patterns 1 and 4, a SNP was counted as correlated with exon-skipping if a 1.5× fold increase in ISO1 CPM was observed from the tumor sample to the matched normal sample, *i.e.* ($\frac{tumor\ ISO1\ CPM}{normal\ ISO1\ CPM} \geq 1.5$). Similarly, for ASE SNPs with Patterns 2 and 5, a SNP was counted as correlated with exon-skipping if a 1.5x fold increase in ISO1 CPM was observed from normal to tumor, *i.e.* ($\frac{normal\ ISO1\ CPM}{tumor\ ISO1\ CPM} \geq 1.5$). For ASE SNPs with Patterns 3 and 6, evidence of exon-skipping was necessary in both samples, *i.e.* (normal ISO1 CPM > 0,tumor ISO1 CPM > 0).

Splice site mutations

Exonic regions of splice site motifs were first defined prior to examining ASE SNPs that could be contributing to the observed alternative splicing. These regions were defined as two bases downstream of the acceptor site (AG) and three bases upstream (5'-ward) of the donor site (GT). Strand information was taken into account while defining these exonic regions for genes on the minus strand. Using BEDTools, ASE SNPs were intersected with the specified exonic regions.

Antisense RNA

Detecting antisense RNA requires the alignment to be performed with reads that have strand information available. The sequence alignment map (BAM) files available on TCGA are missing such information as unstranded library kits were used to generate the reads. The CAFE [12] pipeline predicts the orientation of the unstranded reads using Markov chain models coupled with maximum likelihood estimation given a reference BAM file generated from strand-specific RNA-Seq reads. For the nine patients (3 LUAD: TCGA-44-6776, TCGA-50-5932, TCGA-55-6984; 3 BRCA: TCGA-BH-A0B3, TCGA-BH-A0BW, TCGA-BH-A0DT; 3 HNSC: TCGA-CV-7255, TCGA-CV-7416, TCGA-CV-6959), BAMs consisting of reads with predicted directions were generated using the pipeline and three cell lines with stranded RNA-Seq reads available for each cancer type (LUAD: HCC78 - SRR2050924; BRCA: MCF7 - SRR5048141; HNSC: neuroblastoma-derived cell line – SRR4787038).

Regions where antisense RNA could interfere with the splicing of an exon were determined using the canonical splicing motif for *Homo sapiens* [13] along with a gene's strand. (+ strand: acceptor= -40bp AG +2bp, donor= -3bp GT +5bp; - strand: acceptor= -2bp AG +40bp, donor= -5bp GT +3bp). Using BEDTools, the reads that intersected these regions on the opposite strand of the coding gene were quantified in order to estimate the number of antisense reads. The change in the number of antisense reads mapping to the splicing motif of genes between normal and tumor samples was quantified as a fold change in antisense expression. The read counts were normalized with CPM, using the total number of reads to account for sequencing depth.

REFERENCES

1. 1000 Genomes Project Consortium, Auton A, Brooks LD, Durbin RM, Garrison EP, Kang HM, Korbel JO, Marchini JL, McCarthy S, McVean GA, Abecasis GR. A

global reference for human genetic variation. Nature. 2015; 526:68–74. https://doi.org/10.1038/nature15393. [PubMed]

2. Li H, Handsaker B, Wysoker A, Fennell T, Ruan J, Homer N, Marth G, Abecasis G, Durbin R, 1000 Genome Project Data Processing Subgroup. The Sequence Alignment/Map format and SAMtools. Bioinformatics. 2009; 25:2078–2079. https://doi.org/10.1093/bioinformatics/btp352. [PubMed]
3. Van der Auwera GA, Carneiro MO, Hartl C, Poplin R, Del Angel G, Levy-Moonshine A, Jordan T, Shakir K, Roazen D, Thibault J, Banks E, Garimella KV, Altshuler D, et al. From FastQ data to high confidence variant calls: the Genome Analysis Toolkit best practices pipeline. Curr Protoc Bioinformatics. 2013; 43:11.10.1–33. https://doi.org/10.1002/0471250953.bi1110s43. [PubMed]
4. Delaneau O, Marchini J, Zagury JF. A linear complexity phasing method for thousands of genomes. Nat Methods. 2011; 9:179–181. https://doi.org/10.1038/nmeth.1785. [PubMed]
5. Bansal V, Bafna V. HapCUT: an efficient and accurate algorithm for the haplotype assembly problem. Bioinformatics. 2008; 24:153–159. https://doi.org/10.1093/bioinformatics/btn298. [PubMed]
6. GTEx Consortium. The Genotype-Tissue Expression (GTEx) project. Nat Genet. 2013; 45:580–585. https://doi.org/10.1038/ng.2653. [PubMed]
7. Purcell S, Neale B, Todd-Brown K, Thomas L, Ferreira MA, Bender D, Maller J, Sklar P, de Bakker PI, Daly MJ, Sham PC. PLINK: a tool set for whole-genome association and population-based linkage analyses. Am J Hum Genet. 2007; 81:559–575. https://doi.org/10.1086/519795. [PubMed]
8. McLaren W, Gil L, Hunt SI, Riat HS, Ritchie GR, Thormann A, Flicek P, Cunningham F. The Ensembl Variant Effect Predictor. Genome Biol. 2016; 17:122. https://doi.org/10.1186/s13059-016-0974-4. [PubMed]
9. Gadin JR, van't Hooft FM, Eriksson P, Folkersen L. AllelicImbalance: an R/bioconductor package for detecting, managing, and visualizing allele expression imbalance data from RNA sequencing. BMC Bioinformatics. 2015; 16:194. https://doi.org/10.1186/s12859-015-0620-2. [PubMed]
10. Shen R, Seshan VE. FACETS: Allele-specific copy number and clonal heterogeneity analysis tool estimates for high-throughput dna sequencing. Nucleic Acids Res. 2016; 44:e131. https://doi.org/10.1093/nar/gkw520. [PubMed]
11. Kahles A, Lehmann KV, Toussaint NC, Huser M, Stark SG, Sachsenberg T, Stegle O, Kohlbacher O, Sander C, Cancer Genome Atlas Research Network, Ratsch G. Comprehensive analysis of alternative splicing across tumors from 8,705 patients. Cancer Cell. 2018; 34:211–224. https://doi.org/10.1016/j.ccell.2018.07.001. [PubMed]
12. You BH, Yoon SH, Nam JW. High-confidence coding and noncoding transcriptome maps. Genome Res. 2017; 27:1050–1062. https://doi.org/10.1101/gr.214288.116. [PubMed]
13. Stephens RM, Schneider TD. Features of spliceosome evolution and function inferred from an analysis of the information at human splice sites. J Mol Biol. 1992; 228:1124–1136. https://doi.org/10.1016/0022-2836(92)90320-J.

Supplementary Table 1: Percent of genes displaying ASE in 233 TCGA patients

	Pattern	% Total Genes	% All COSMIC	% TSG	% Oncogene	% Fusion
BRCA	1	9.6	9.3	9.1	8.3	9.3
	2	0.5	0.5	0.4	0.3	0.7
	3	0.0	0.0	0.0	0.0	0.0
	4	3.4	3.3	2.8	3.4	3.7
	5	2.7	2.2	2.1	2.5	2.1
	6	0.1	0.1	0.0	0.0	0.1
	No ASE	83.7	84.6	85.6	85.5	84.0
HNSC	1	12.4	12.5	11.1	13.6	13.1
	2	0.4	0.3	0.1	0.6	0.6
	3	0.1	0.1	0.1	0.0	0.2
	4	4.2	4.1	4.5	3.9	3.8
	5	2.6	2.0	2.0	2.1	1.7
	6	0.1	0.2	0.1	0.1	0.3
	No ASE	80.3	80.9	82.1	79.7	80.3
LUAD	1	13.4	13.1	13.2	12.5	14.0
	2	0.3	0.3	0.1	0.4	0.4
	3	0.0	0.1	0.0	0.0	0.1
	4	5.2	5.7	5.6	5.5	5.8
	5	1.7	1.3	1.4	1.6	1.4
	6	0.1	0.0	0.0	0.1	0.0
	No ASE	79.3	79.5	79.6	80.0	78.3
THCA	1	3.3	2.9	2.8	3.3	3.8
	2	0.3	0.3	0.1	0.2	0.6
	3	0.0	0.0	0.0	0.0	0.0
	4	0.5	0.5	0.2	0.5	1.0
	5	2.6	2.2	2.6	2.3	2.2
	6	0.0	0.0	0.0	0.0	0.0
	No ASE	93.3	94.1	94.3	93.8	92.4

Supplementary Table 2: DNA-sequencing (DNA-seq) and RNA-sequencing (RNA-seq) data sources for the nine TCGA patients analyzed for mechanism of ASE in this study

ID	TCGA Barcode	Cancer Type	Sex	Age	Race	Sample Typea	Seq Depth b	Read Len. c
Breast 1	TCGA-BH-A0B3-11B-21D-A128-09	Breast Invasive Carcinoma	F	53	White	NT-G	42.4	100
	TCGA-BH-A0B3-11B-21R-A089-07					NT-R	5.5	50
	TCGA-BH-A0B3-11B-21W-A100-09					NT-X	8.1	100
	TCGA-BH-A0B3-01A-11D-A128-09					TP-G	40.2	100
	TCGA-BH-A0B3-01B-21R-A089-07					TP-R	5.4	50
	TCGA-BH-A0B3-01A-11W-A071-09					TP-X	10.5	100
Breast 2	TCGA-BH-A0BW-11A-12D-A314-09		F	71	Black	NT-G	54.1	100
	TCGA-BH-A0BW-11A-12R-A115-07					NT-R	7	50
	TCGA-BH-A0BW-11A-12D-A10Y-09					NT-X	14.7	100
	TCGA-BH-A0BW-01A-11D-A10Y-09					TP-G	46.1	100
	TCGA-BH-A0BW-01A-12R-A115-07					TP-R	7.3	50
	TCGA-BH-A0BW-01A-11D-A10Y-09					TP-X	15.9	100
Breast 3	TCGA-BH-A0DT-11A-12D-A12B-09		F	41	White	NT-G	63.3	100
	TCGA-BH-A0DT-11A-12R-A12D-07					NT-R	7.7	50
	TCGA-BH-A0DT-11A-12D-A12B-09					NT-X	22.7	100
	TCGA-BH-A0DT-01A-21D-A12B-09					TP-G	79.9	100
	TCGA-BH-A0DT-01A-21R-A12D-07					TP-R	6.6	50
	TCGA-BH-A0DT-01A-21D-A12B-09					TP-X	21.7	100
Head 1	TCGA-CV-7255-11A-01D-2276-10	Head and Neck Squamous Cell Carcinoma	F	32	White	NT-G	6.9	101
	TCGA-CV-7255-11A-01R-2016-07					NT-R	7.5	48
	TCGA-CV-7255-11A-01D-2012-08					NT-X	27.3	76
	TCGA-CV-7255-01A-11D-2276-10					TP-G	5.8	101
	TCGA-CV-7255-01A-11R-2016-07					TP-R	7.1	48
	TCGA-CV-7255-01A-11D-2012-08					TP-X	28.9	76
Head 2	TCGA-CV-7416-11A-01D-2334-08		F	29	White	NT-G	7.7	101
	TCGA-CV-7416-11A-01R-2081-07					NT-R	5.9	48
	TCGA-CV-7416-11A-01D-2078-08					NT-X	23.9	76
	TCGA-CV-7416-01A-11D-2334-08					TP-G	28.6	101
	TCGA-CV-7416-01A-11R-2081-07					TP-R	6	48
	TCGA-CV-7416-01A-11D-2078-08					TP-X	25.0	76
Head 3	TCGA-CV-6959-11A-01D-1911-02		M	48	White	NT-G	38.3	51
	TCGA-CV-6959-11A-01R-1915-07					NT-R	8.5	48
	TCGA-CV-6959-11A-01D-1912-08					NT-X	26.8	76
	TCGA-CV-6959-01A-11D-1911-02					TP-G	31.4	51
	TCGA-CV-6959-01A-11R-1915-07					TP-R	6.6	48
	TCGA-CV-6959-01A-11D-1912-08					TP-X	28.0	76

Lung 1	TCGA-44-6776-11A-01D-1853-02	Lung Adenocarcinoma	F	60	White	NT-G	38.9	51
	TCGA-44-6776-11A-01R-1858-07					NT-R	5.4	48
	TCGA-44-6776-11A-01D-1855-08					NT-X	29.1	76
	TCGA-44-6776-01A-11D-1853-02					TP-G	6.9	51
	TCGA-44-6776-01A-11R-1858-07					TP-R	7.4	48
	TCGA-44-6776-01A-11D-1855-08					TP-X	30.8	76
Lung 2	TCGA-50-5932-11A-01D-1753-08		M	75	White	NT-G	34.6	101
	TCGA-50-5932-11A-01R-1755-07					NT-R	4.2	48
	TCGA-50-5932-11A-01D-1753-08					NT-X	29.3	76
	TCGA-50-5932-01A-11D-1753-08					TP-G	44.5	101
	TCGA-50-5932-01A-11R-1755-07					TP-R	7.4	48
	TCGA-50-5932-01A-11D-1753-08					TP-X	33.6	76
Lung 3	TCGA-55-6984-11A-01D-1945-08		F	NA	White	NT-G	36.2	101
	TCGA-55-6984-11A-01R-1949-07					NT-R	4.9	48
	TCGA-55-6984-11A-01D-1945-08					NT-X	23.1	76
	TCGA-55-6984-01A-11D-1945-08					TP-G	41	101
	TCGA-55-6984-01A-11R-1949-07					TP-R	5.2	48
	TCGA-55-6984-01A-11D-1945-08					TP-X	17.8	76

[a]NT-G=Normal tissue WGS, NT-R=Normal tissue RNA-seq, NT-X=Normal tissue WXS, TP-G=Tumor primary WGS, TP-R=Tumor primary RNA-seq, TP-X=Tumor primary WXS; [b]Average coverage; [c]Read length measured in base pairs

Supplementary Table 3: ASE genes possibly explained by eQTLs or differential methylation of CpG islands

**Patient**	**Total ASE Genes**	**ASE Genes w phased eQTL genotypes that explain Pattern**	**Percentage of ASE putatively explained by eQTLs**	**ASE Genes w beta value | fold change | > 1.3**	**Percentage of ASE correlated**
Breast 1	845	8	0.9%	113	13.4
Breast 2	834	6	0.7%	25	3.0
Breast 3	296	5	1.7%	9	3.4
Head & Neck 1	447	*eQTLs not available*		87	19.5
Head & Neck 2	NA	*eQTLs not available*		*Methylation data not available*	
Head & Neck 3	540	*eQTLs not available*		65	12.0
Lung 1	458	25	5.4%	*Methylation data not available*	
Lung 2	131	1	5.1%	15	13.0
Lung 3	201	4	5.4%	*Methylation data not available*	
**Total**	3,752	49	**1.8%**	317	**10.2**

Supplementary Table 4: LoF mutations in random samplings of non-TSGs from 1KGP

Iteration	Number of damaging mutations	Average number of damaging mutations Per Gene
Iteration1	2204	8.0
Iteration2	2460	9.7
Iteration3	2518	9.5
Iteration4	2371	8.9
Iteration5	2284	9.0

Supplementary Table 5: LoF mutations within TSGs in normal samples from TCGA

	Number of damaging mutations	Average number of damaging mutations Per Gene
**BRCA**	89	1.6
**HNSC**	38	1.4
**THCA**	44	1.4
**LUAD**	40	1.5

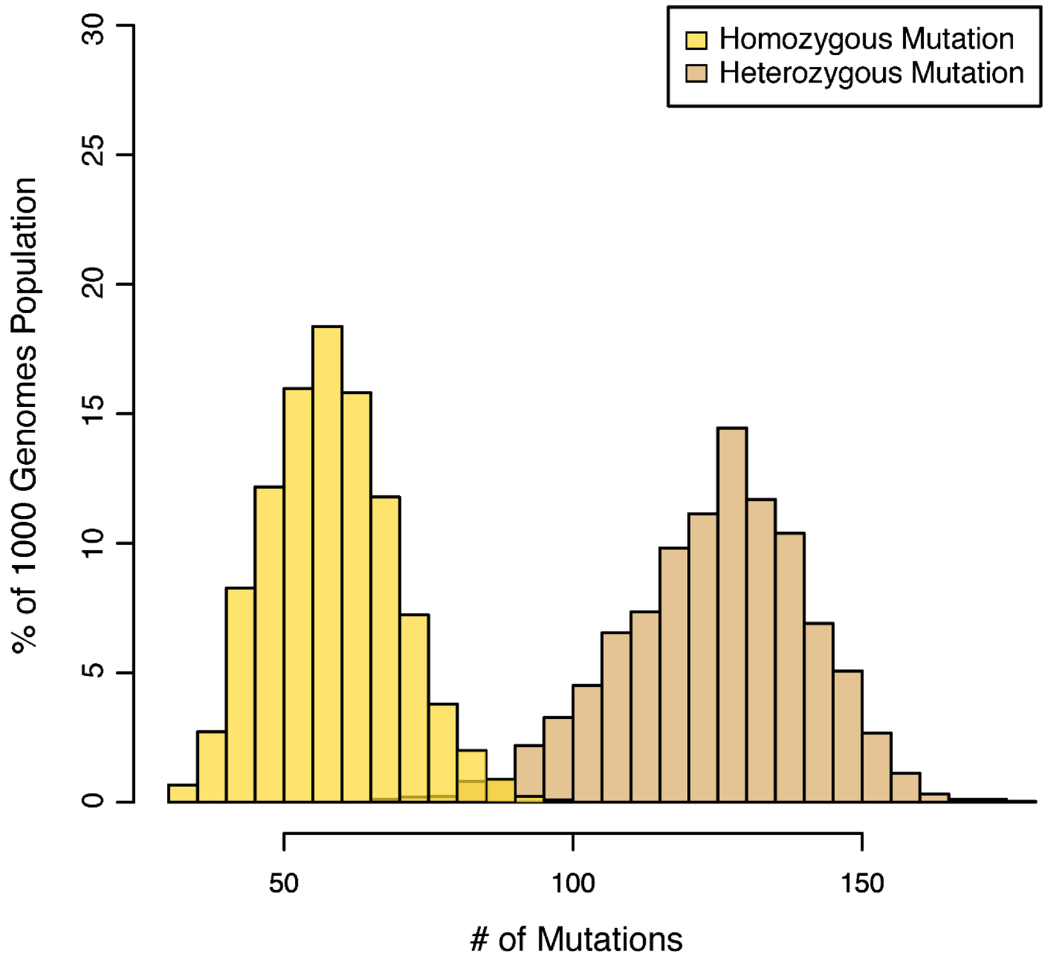

Supplementary Figure 1: Distribution of COSMIC census mutations in the 1KGP. The distribution of cancer associated mutations (all coding mutations in COSMIC Census Genes) within the 1KGP.

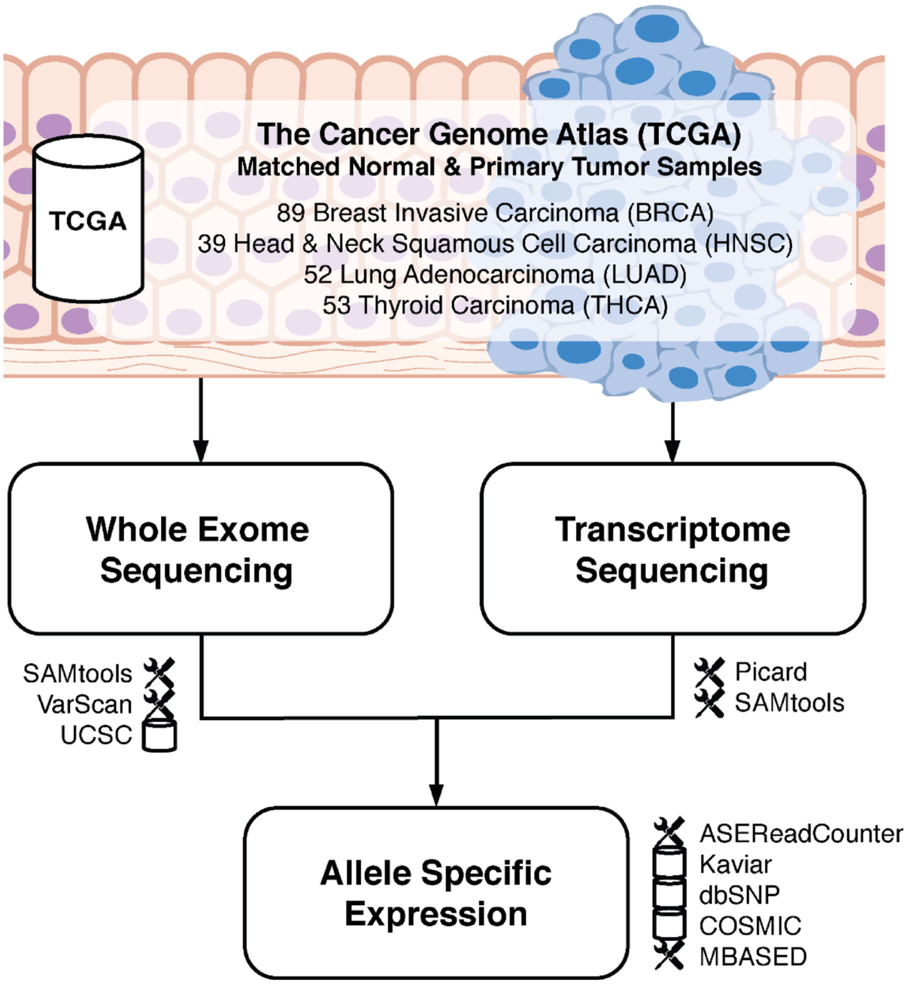

Supplementary Figure 2: ASE workflow used in this study. Matched normal and primary tumor samples for four cancer types were analyzed for allele-specific expression using whole exome (DNA-seq) and transcriptome (RNA-seq) data. DNA-seq data were used to identify heterozygous sites in the exome and RNA-seq data were analyzed to compare expression of reference vs alternative alleles at those sites. The core bioinformatics databases and tools used for each stage of the pipeline are indicated.

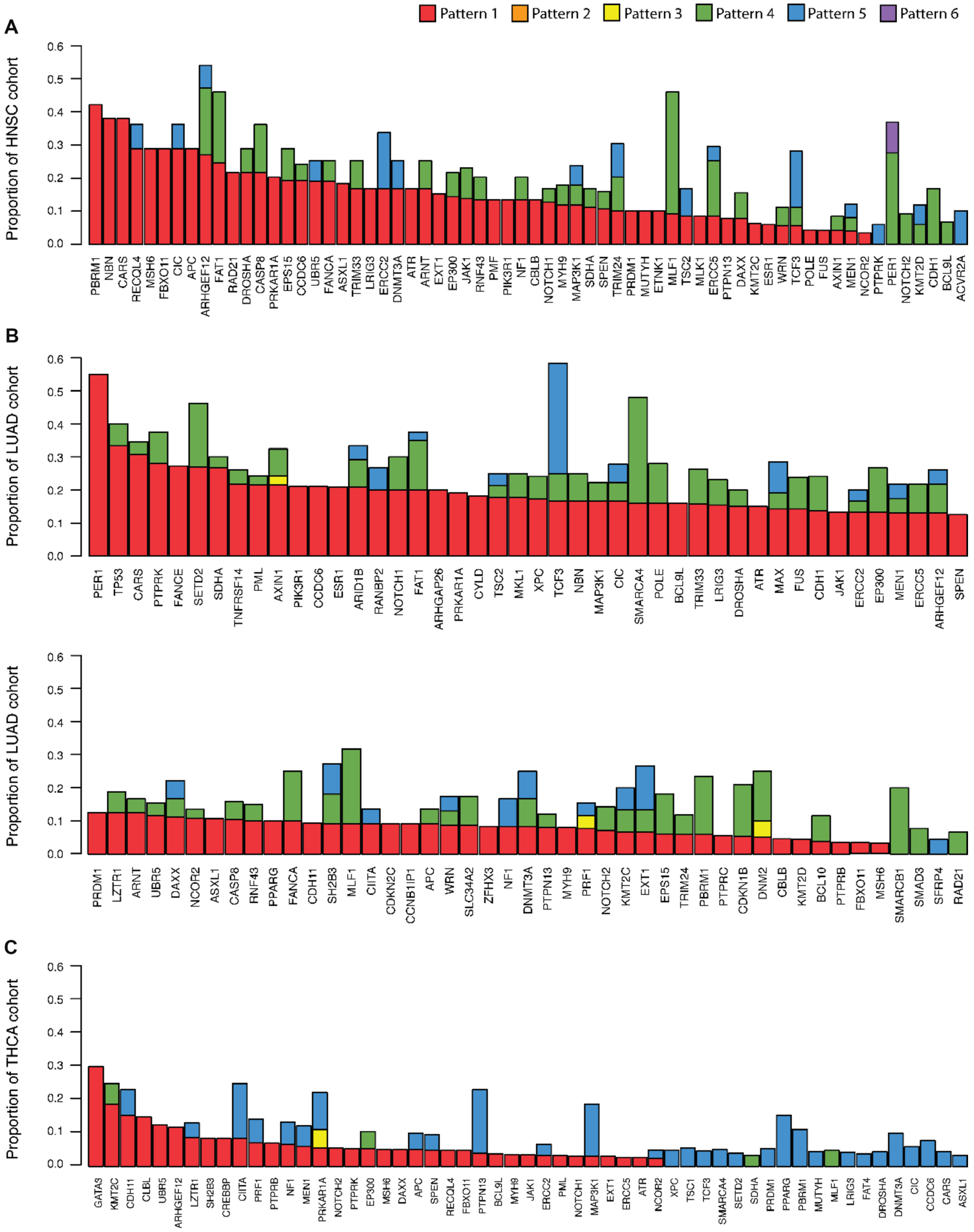

Supplementary Figure 3: Frequency of ASE in tumor suppressor genes. Gene level ASE was computed as described in the Materials and Methods section. (**A**) The proportion of head and neck cancer patients with ASE in 64 TSGs; (**B**) the proportion of lung cancer patients with ASE in 89 TSGs; and (**C**) the proportion of thyroid cancer patients with ASE in 55 TSGs.

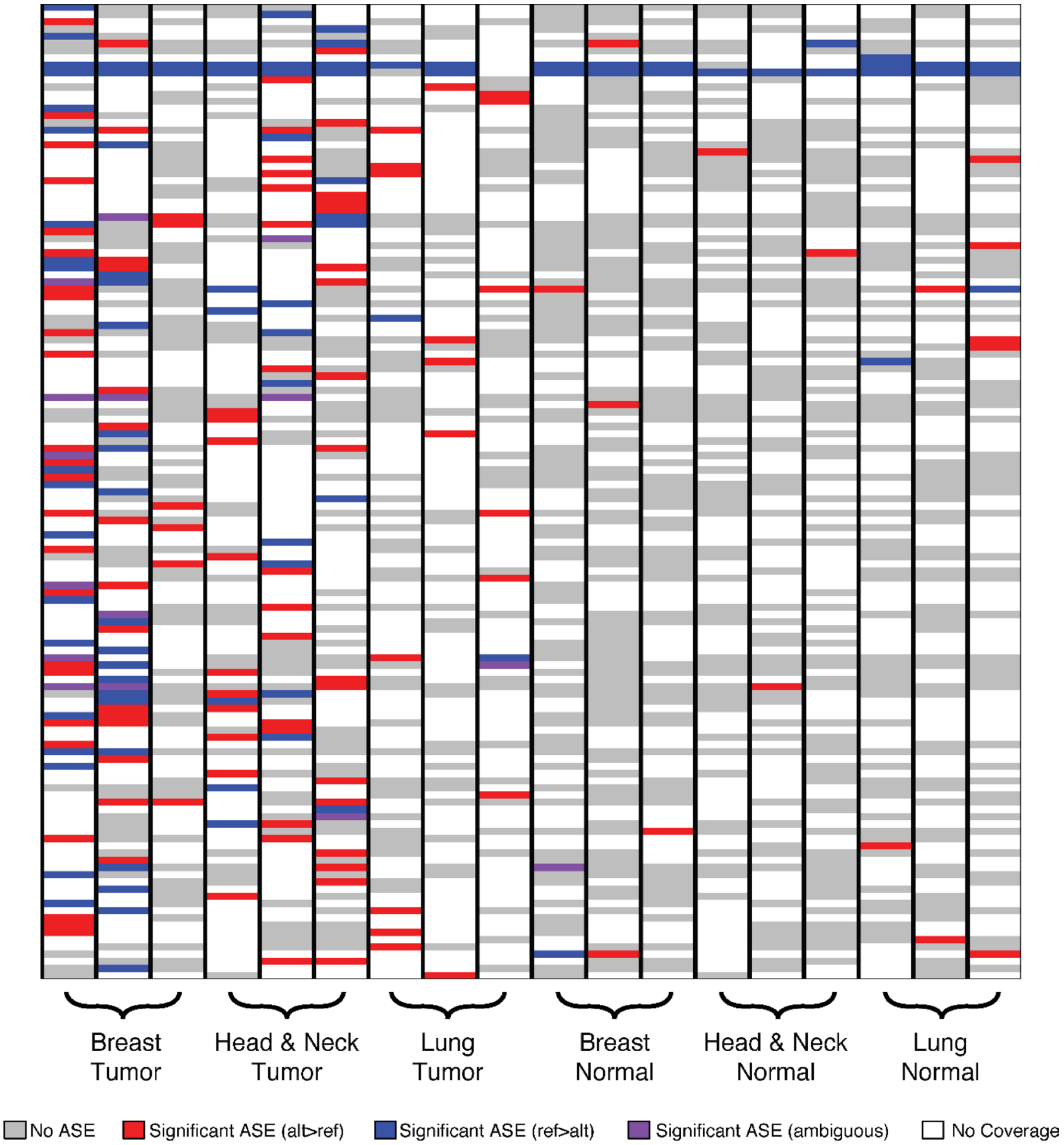

Supplementary Figure 4: Heatmap of COSMIC genes across nine patients analyzed for mechanism of ASE. Significant ASE ($P < 0.05$) genes are shown in red (alt > ref) and blue (ref > alt). Note some genes have significant ASE, however their direction is ambiguous (purple) because of an equal number of reference and alternative SNPs on each pseudo-haplotype.

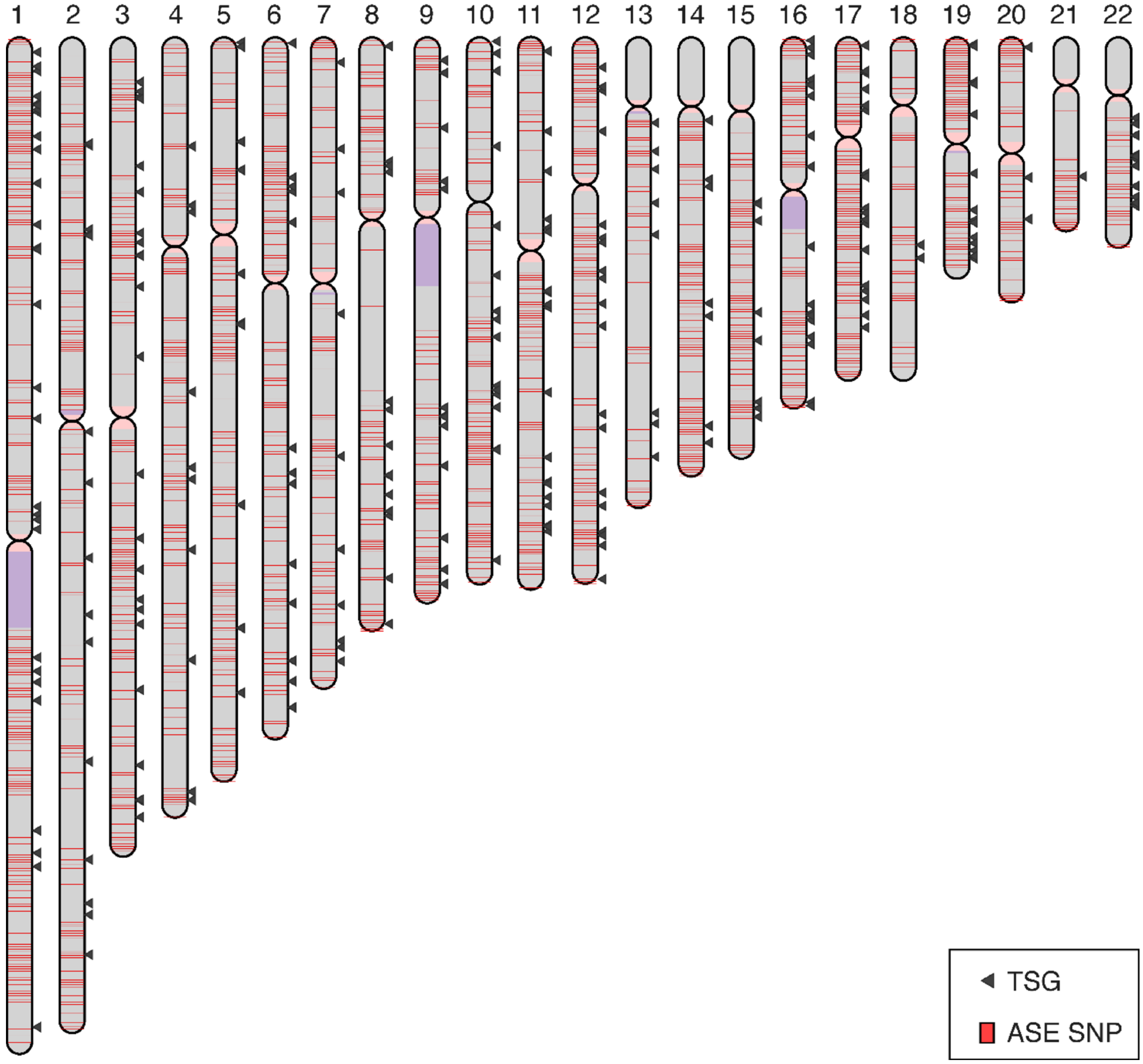

Supplementary Figure 5: Distribution of ASE SNPs in the genome from nine patients studied for mechanism. Genome ideogram showing locations of ASE SNPs marked in red; TSGs denoted with triangle. Chromosomes are rendered with pink centromeres and purple heterochromatin bands.

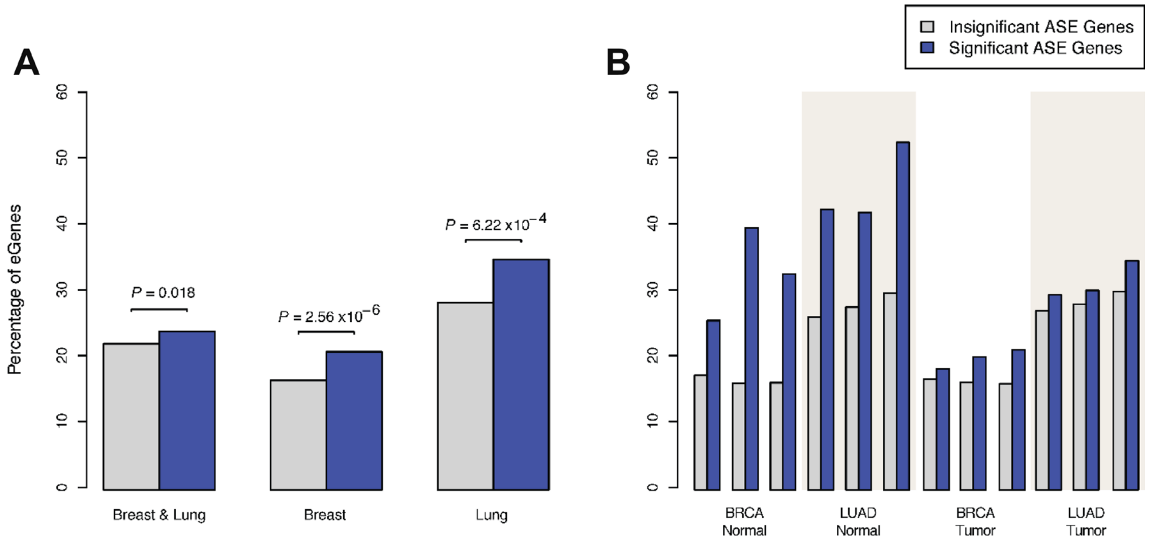

Supplementary Figure 6: ASE eGenes. Heterozygous eQTLs associated with protein coding genes (eGenes) were identified in patient samples using whole genome sequencing (WGS). (**A**) Percentages of ASE genes associated with eQTLs in breast and lung cancer patient samples. (**B**) Percentages of ASE genes associated with eQTLs broken down by individual patient sample.

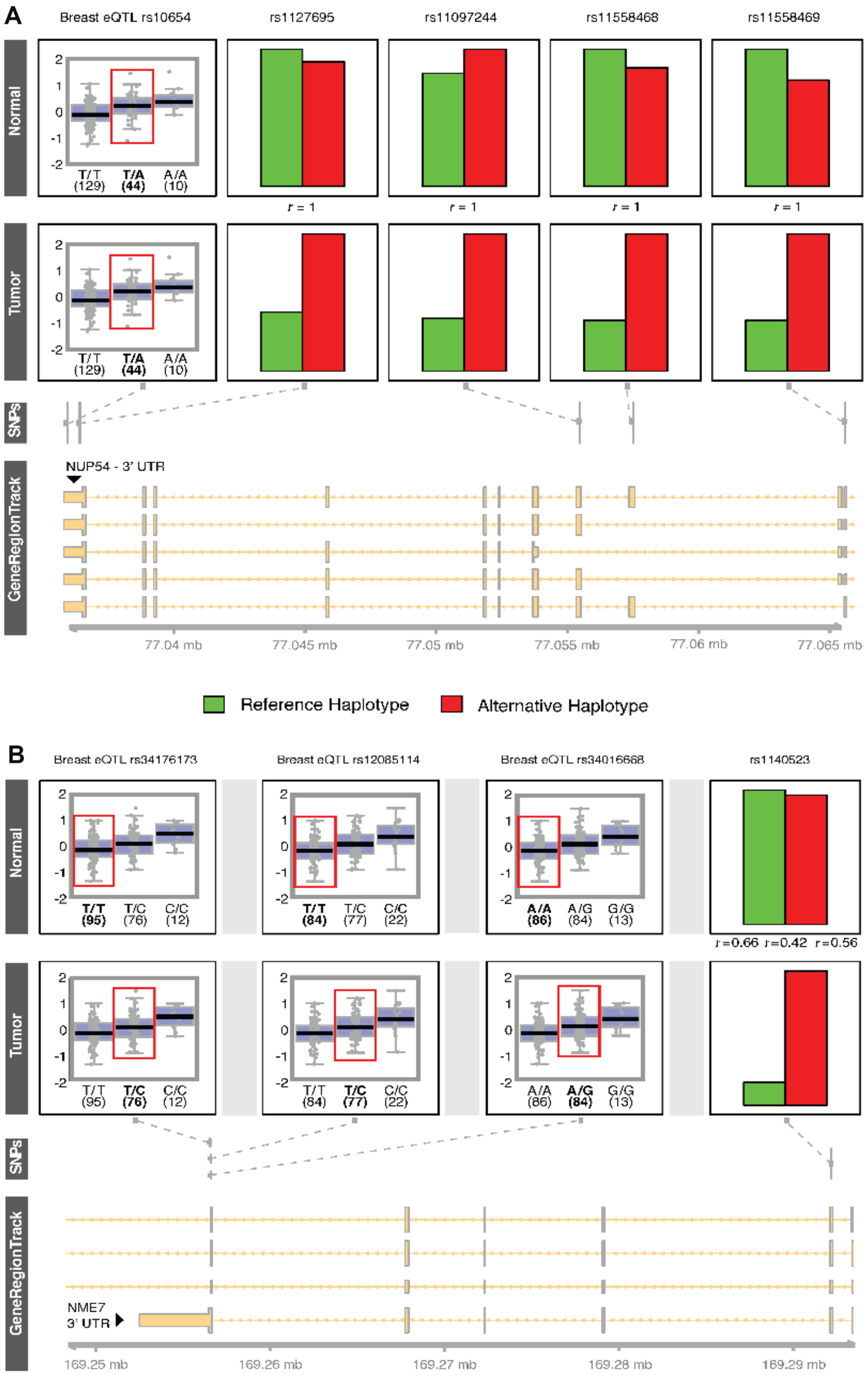

Supplementary Figure 7: eQTLs in cis and trans with ASE genes. Specific genomic regions spanning eQTLs and ASE SNPs were plotted using AllelicImbalance as described in the Materials and Methods. The top panels contain GTEx single tissue eQTL box plots and the relative expression of alleles for linked SNPs. The grey lines in the middle panel show where the SNP locations lie in relation to the appropriate genome track shown beneath in yellow. (**A**) A heterozygous eQTL present in the 3′ UTR of NUP54 in both the normal and tumor samples of a breast invasive carcinoma patient. (**B**) Three eQTLs present downstream from NME7, which are homozygous reference in the normal sample and heterozygous in the tumor sample of a breast invasive carcinoma patient.

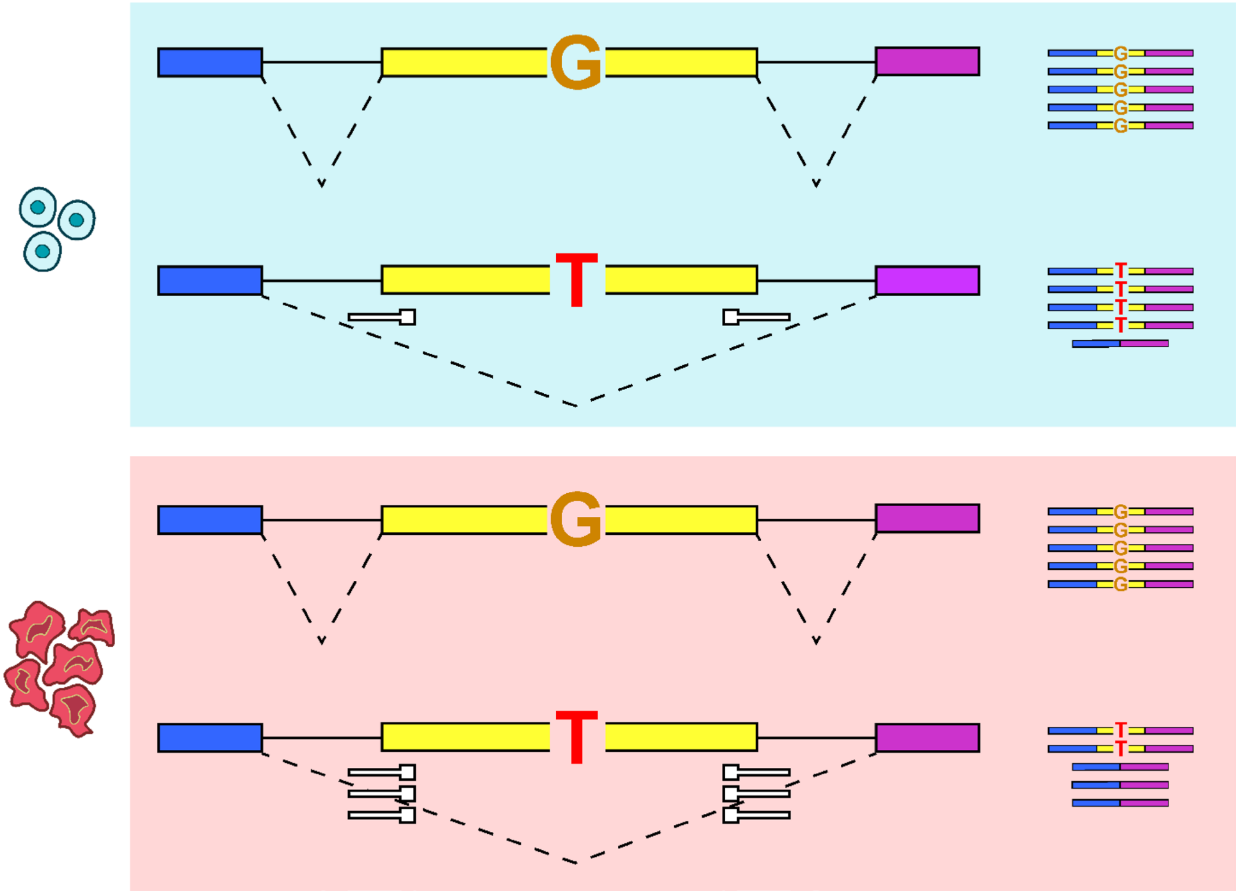

Supplementary Figure 8: Model for antisense induced allele-specific exon skipping and its contribution to ASE. This cartoon example illustrates how antisense RNA could induce allele-specific exon skipping resulting in ASE Pattern 1. The top panel shows little antisense RNA ( ) mapping to splice sites, thus inducing little amounts of exon skipping of the "T" allele and negligible ASE in the normal sample. The bottom panel shows larger quantities of antisense RNA mapping to splice sites, inducing exon-skipping of the "T" allele and leading to substantial ASE in the tumor.

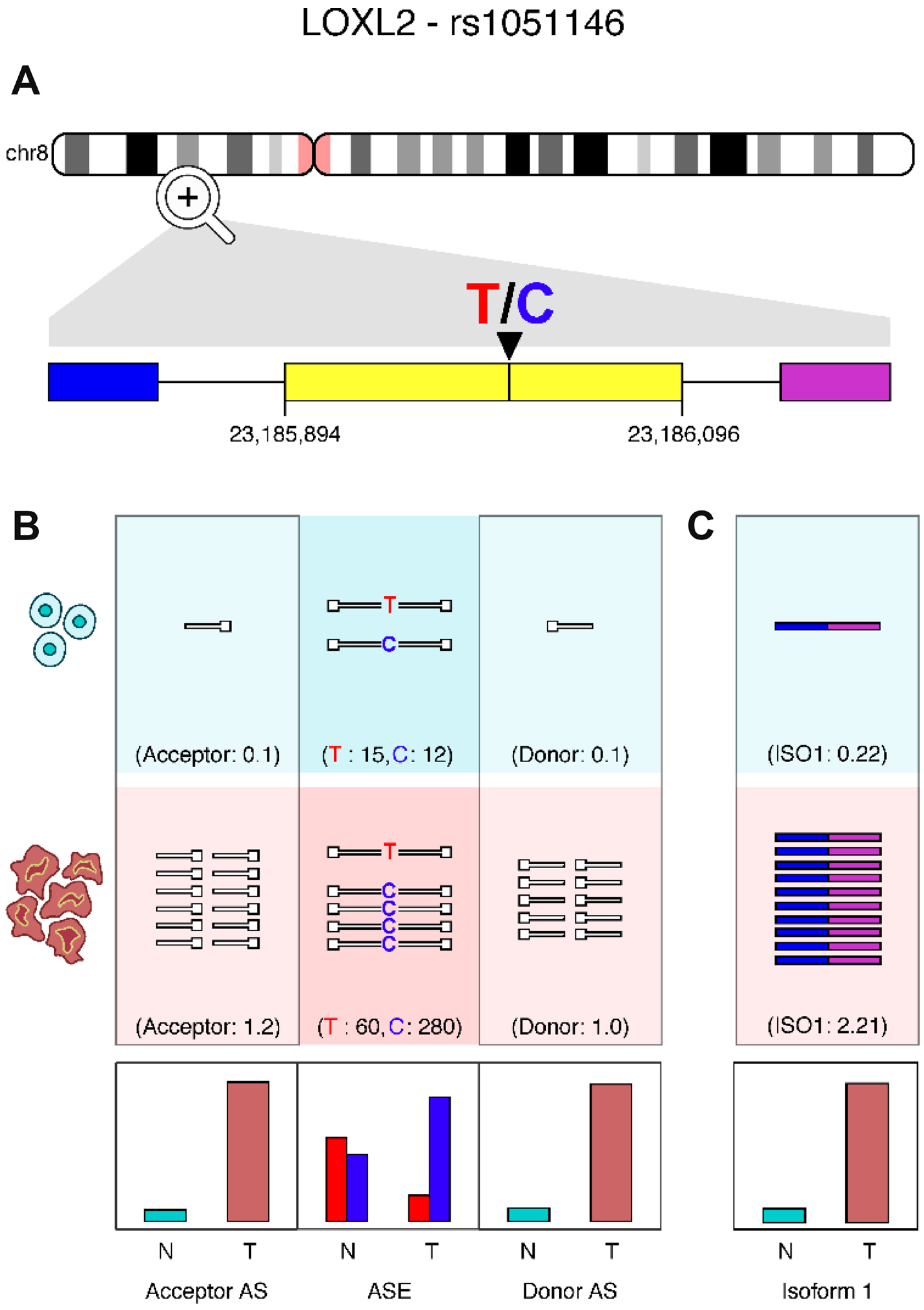

Supplementary Figure 9: LOXL2 exon skipping correlates with ASE in a breast adenocarcinoma patient. (**A**) An exon skipping event in exon 6 of TNC in a breast cancer patient (TCGA-BH-A0B3). (**B**) Antisense reads ( ) mapping to donor and acceptor sites are quantified, alongside the ASE locus within the exon. (**C**) Quantification of reads supporting the isoform missing exon 6. Relative expression plots are shown for antisense RNA, ASE and isoforms below.

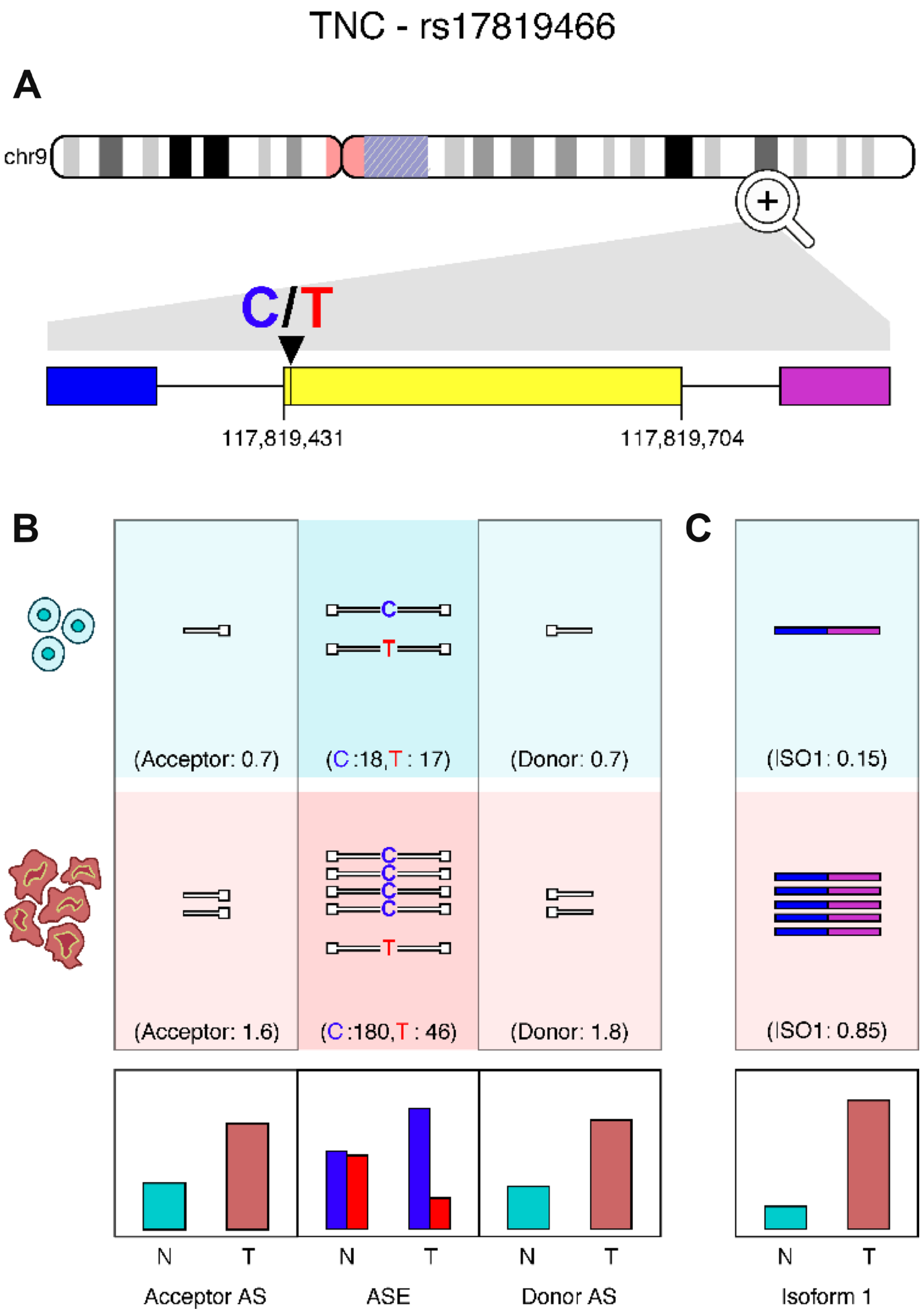

Supplementary Figure 10: TNC exon skipping correlates with ASE in a breast adenocarcinoma patient. (**A**) An exon skipping event in exon 15 of TNC in a breast cancer patient (TCGA-BH-A0DT). (**B**) Antisense reads ( ) mapping to donor and acceptor sites are quantified, alongside the ASE locus within the exon. (**C**) Quantification of reads supporting the isoform missing exon 15. Relative expression plots are shown for antisense RNA, ASE and isoforms below.

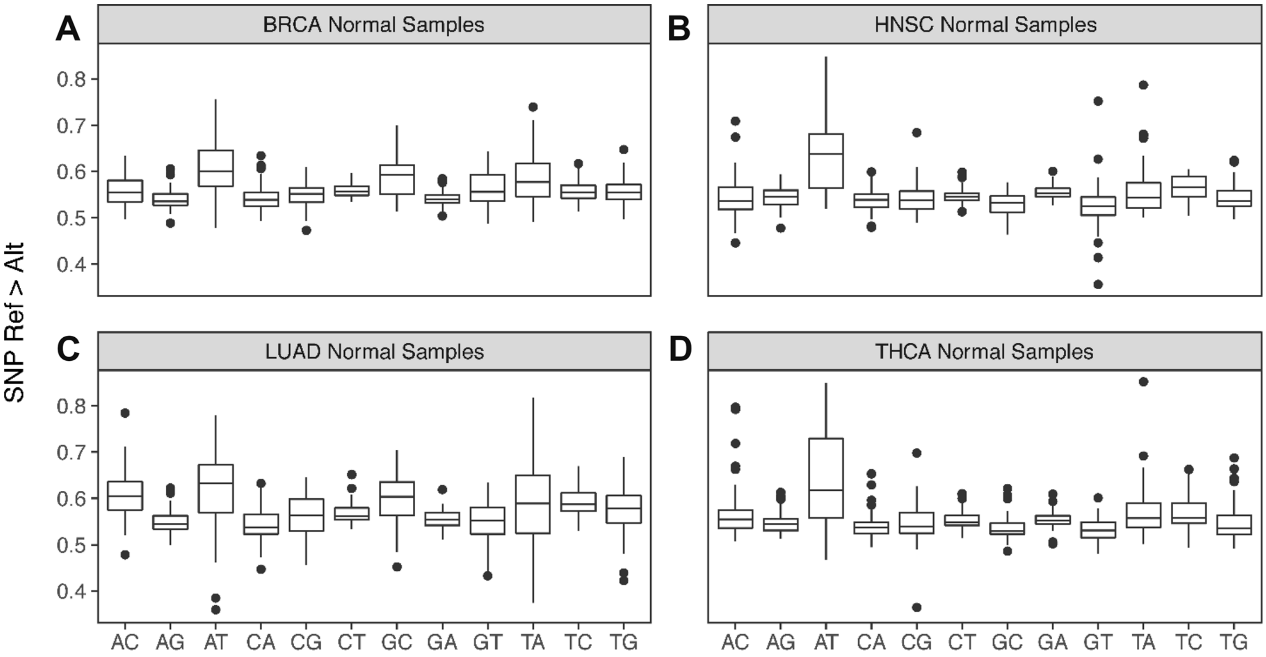

Supplementary Figure 11: Allelic ratios for all possible nucleotide combinations. Distribution of proportion of sites where reference allele count is greater than alternative allele count in normal samples for (**A**) breast cancer patients, (**B**) head and neck cancer patients, (**C**) lung cancer patients, and (**D**) thyroid cancer patients.

Supplementary Dataset 1: Loss-of-function COSMIC mutations identified in 1000 Genomes Population. See Supplementary Dataset 1

Supplementary Dataset 2: ASE SNPs intersecting canonical splice motifs. See Supplementary Dataset 2

Supplementary Dataset 3: TCGA patient metadata. See Supplementary Dataset 3

Supplementary Dataset 4: TCGA sample metadata. See Supplementary Dataset 4
